# Synthesis‐Driven Functionality in High‐Entropy Materials

**DOI:** 10.1002/smll.202501703

**Published:** 2025-09-30

**Authors:** Anurag Khandelwal, George Mathew, Subramshu Bhattacharya, Alexander Colsmann, Gabriel Cadilha Marques, Miriam Botros, Florian Strauss, Jiangyuan Xing, John Silvister Raju, Arivazhagan Ponnusamy, Jasmin Aghassi‐Hagmann, Torsten Brezesinski, Simon Schweidler, Ben Breitung

**Affiliations:** ^1^ Institute of Nanotechnology Karlsruhe Institute of Technology (KIT) Kaiserstr. 12 76131 Karlsruhe Germany; ^2^ Department of Metallurgical and Materials Engineering Nano Functional Materials Technology Centre (NFMTC) Indian Institute of Technology Madras Chennai 600036 India; ^3^ Material Research Center for Energy Systems Karlsruhe Institute of Technology (KIT) Kaiserstr. 12 76131 Karlsruhe Germany; ^4^ Department of Mechanical and Automobile Engineering Christ University Bengaluru India

**Keywords:** electronic applications, energy applications, high‐entropy materials, high‐entropy oxides, synthesis techniques

## Abstract

Since their discovery in 2015, high‐entropy oxides have introduced a paradigm shift in materials science, unveiling a class of compounds with exceptional structural and functional versatility. These high‐entropy materials (HEMs) offer exciting opportunities as next‐generation alternatives to conventional materials, owing to the synergistic interplay of multiple principal elements that results in enhanced stability, tunability, and multifunctionality. Their unique atomic configurations enable the design of materials with high surface areas and abundant active sites for catalysis, mechanically robust structures for energy storage, or tunable band gaps for electronic and optoelectronic devices. However, the vast compositional space of HEMs presents both a challenge and an opportunity. Meaningful property design requires a deep understanding of how synthesis routes influence structure–property relationships. In this review, a comprehensive overview of established and emerging synthesis strategies for HEMs, focusing on how each method affects resulting structural, electronic, electrochemical, and optical characteristics, is provided. Key process parameters that can be tailored to optimize material performance are highlighted. Additionally, the accelerating role of high‐throughput synthesis and characterization in navigating the design space of high‐entropy systems is discussed. By systematically connecting synthesis, structure, and function, this review aims to guide the rational design of HEMs for energy applications and beyond.

## Graphical abstract

This review explores how synthesis techniques shape the structure, morphology, and functional properties of high‐entropy materials for energy and electronic applications. By comparing diverse methods through nine key criteria and linking them to material performance, the work offers a practical framework to guide synthesis selection for targeted functionality in advanced devices.

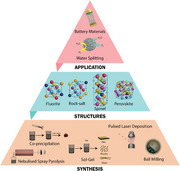

## 1 Introduction

High‐entropy materials (HEMs) represent an emerging and rapidly expanding class of compounds that exhibit outstanding potential across a wide range of disciplines, particularly in the fields of energy conversion, storage, and electronic applications. Their appeal stems from their unique combination of compositional complexity, structural disorder, and exceptional tunability, which enables unprecedented property engineering. In contrast to conventional materials with one or two primary elements, HEMs incorporate multiple principal components, typically five or more, in near‐equiatomic ratios. This compositional richness results in complex interactions at the atomic scale, opening the door to materials with novel properties that are unattainable through classical design strategies. Their high chemical flexibility and adaptability to external influences make them particularly well‐suited for applications ranging from catalysis and energy storage to semiconductors and sensors.

Formally, HEMs are defined either by having at least five distinct elements with atomic fractions between 5 and 35%, or by achieving a configurational entropy exceeding 1.5 *R* within a stable, single‐phase crystal structure.^[^ ^1^, ^2^, ^3^, ^4^ ^]^ Since their properties are often highly sensitive to composition, the potential for targeted design is vast. The concept was originally introduced through high‐entropy alloys (HEAs), with the Cantor alloy (CoCrMnNiFe) serving as a prominent example.^[^ ^1^ ^]^ The high‐entropy concept was soon extended to ionic compounds, especially ceramics, leading to a wide range of multicomponent systems such as high‐entropy oxides, fluorides, oxyfluorides, sulfides, and beyond.^[^ ^5^, ^6^ ^]^ More recently, the approach has been applied to structurally complex systems such as metal‐organic frameworks (MOFs) and MXenes, where compositional design is leveraged to tune mechanical, morphological, electronic, and catalytic properties.^[^ ^7^, ^8^ ^]^

Among the most promising energy‐relevant HEMs are multicomponent ceramics and MOFs. These materials are increasingly explored for their advantageous properties in batteries, electronics, electrocatalysis, and photocatalysis.^[^ ^9^, ^10^, ^11^ ^]^ Their compositional complexity offers opportunities to fine‐tune key parameters such as ionic conductivity, catalytic activity, stability, or band structure. In catalysis, for instance, activity and selectivity are largely governed by surface morphology and electronic configuration, both of which can be controlled through compositional engineering. Due to their atomic‐level homogeneity, HEMs offer a rich variety of chemically distinct yet uniformly distributed catalytic sites. These environments exhibit subtly varying electronic structures, providing a broad energy landscape for reactions, a feature that distinguishes HEMs as a fundamentally new class of catalytic materials.

The remarkable functional properties of HEMs arise from several core phenomena: cocktail effects, lattice distortions, chemical versatility, and tunable configurational entropy.^[^ ^6^, ^12^ ^]^ Cocktail effects describe the synergistic interactions between multiple cations with diverse oxidation states, electronegativities, and electronic configurations, leading to emergent behaviors, such as the unexpected stabilization of Cu(0) in oxide lattices, or complex redox pathways in mixed‐valence systems.^[^ ^13^, ^14^ ^]^ Lattice distortions, caused by size mismatches between constituent ions, can significantly affect mechanical properties, diffusion kinetics, and electronic structure through altered bond angles and strain fields.^[^ ^15^, ^16^, ^17^ ^]^ The concept of chemical versatility refers to the capacity to replace scarce or toxic elements with more sustainable alternatives, without compromising functionality, an aspect closely tied to the overall tailorability of HEMs.^[^ ^18^ ^]^

The synthesis of HEMs is a challenging endeavor due to the inherent complexity of incorporating multiple elements into a stable single‐phase structure. Parameters such as ionic radii, oxidation states, coordination preferences, and electronic configurations must be carefully balanced to avoid phase segregation or the formation of undesired secondary phases.^[^ ^19^, ^20^ ^]^ Achieving phase‐pure compounds with controlled morphology and stoichiometry often requires meticulous tuning of synthesis conditions, including precursor chemistry, reaction atmosphere, thermal treatment profiles, and solvent environments.

The functional deployment of HEMs also demands that synthesis be aligned with the specific requirements of each application. In many cases, this involves transitioning from bulk powders to well‐defined architectures such as nanostructured surfaces or thin films. For example, the development of uniform thin films without cracks or pinholes is essential for electronic applications, while catalytic materials may benefit from tailored particle sizes and high surface areas. The ability to precisely control morphology, phase formation, and elemental distribution is therefore critical. Importantly, different synthesis routes can yield materials with markedly different structures and properties, even when starting from the same elemental composition.^[^ ^21^ ^]^

Given the intricate coupling between synthesis, structure, and functionality in HEMs, navigating the variety of available synthesis techniques presents a significant challenge. This review provides a comprehensive overview of current synthesis strategies for high‐entropy materials, analyzing their underlying mechanisms, prerequisites, and the extent to which they enable control over composition, phase, and morphology. Emphasis is placed on the connection between synthesis parameters and application‐relevant material properties, with the goal of equipping researchers with the insights needed to design and develop next‐generation HEMs for energy and electronics.

### 1.1 General Considerations for the Synthesis and Integration of High‐Entropy Materials

**Figure 1:**
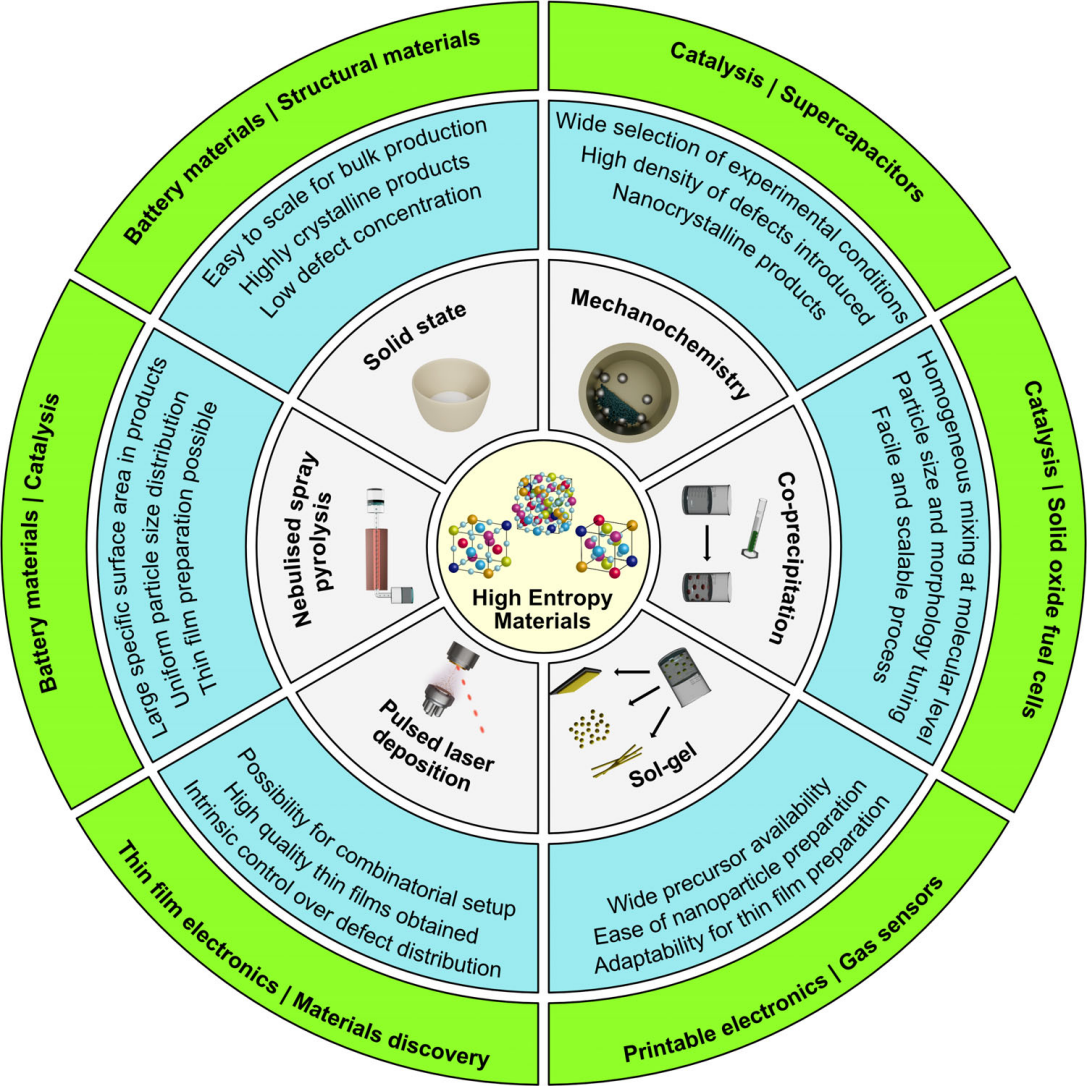
Overview of the various synthesis routes, their advantages, and suggested application areas for high entropy materials.

The synthesis of inorganic powder materials encompasses a wide range of techniques, each with inherent advantages and limitations. The selection of an appropriate method depends on factors such as material composition, required morphology, cost‐efficiency, scalability, and the ability to control physicochemical properties reproducibly. Especially in the context of HEMs, where compositional complexity is intrinsic, synthesis approaches must meet additional criteria: they must ensure homogeneous mixing at the atomic level, stabilize single‐phase structures, and offer tunability with respect to morphology, particle size, and crystallinity. For industrial‐scale production, continuous and scalable processes with stable precursor supply and reproducible product quality are imperative. At the same time, methods must enable application‐specific adjustments, such as the preparation of nanostructured powders for catalysis or energy storage, where surface area and active site accessibility are key performance parameters (see **Figure** **1**).

The synthesis of high‐entropy inorganic materials presents distinct challenges beyond those encountered with simpler multicomponent systems. Most notably, atomic‐scale homogeneity is critical to achieving a high configurational entropy that defines HEMs. The precursor components must be intimately mixed and compatible in terms of oxidation state, coordination behavior, and ionic radius. Even minor deviations can lead to phase separation, particularly during the high‐temperature calcination steps typically required to crystallise ceramic HEMs. These calcination temperatures often exceed 1000 °C, and rapid quenching is frequently necessary to “lock in” the high‐entropy phase and prevent thermodynamic relaxation into multiple, simpler phases during cooling. This is particularly important in systems involving redox‐active transition metals such as Mn, which can exhibit multiple oxidation states and complex spin‐state behavior depending on the thermal history and local environment.^[^ ^22^, ^23^, ^24^, ^25^ ^]^

Beyond powder synthesis, the successful implementation of HEMs in functional devices depends critically on their processability, that is, the ability to structure and integrate the materials into relevant device architectures. This requires not only chemical compatibility with other device components but also control over morphology, film quality, and interfacial properties. For example, thin films prepared via physical vapor deposition techniques such as pulsed laser deposition (PLD) often offer excellent surface uniformity and adhesion but may impose restrictions on the compositional complexity or the achievable crystalline phase due to kinetic limitations. Conversely, powder‐based synthesis routes may yield materials with desirable compositions and phases, but further processing, such as milling, dispersion, or formulation with binders and surfactants, may be required to render them suitable for screen‐printing, spin coating, or inkjet deposition.

The interdependencies between synthesis method, material properties, structuring strategy, and application requirements are therefore highly complex. For instance, a synthesis route that yields high crystallinity may result in particles that are too large for catalytic applications requiring high surface‐to‐volume ratios. Alternatively, methods that produce nanostructured powders may lead to poor phase purity or require additional processing to ensure long‐term stability. Figure 1 schematically illustrates these relationships, highlighting how synthesis conditions influence material composition, size, and morphology, which in turn affect the structuring approach and ultimately the material's suitability for specific applications in electronics or energy.

To navigate this intricate landscape, the following sections provide a detailed overview of major synthesis strategies used in the development of high‐entropy materials. For each approach, we discuss the relevant physical and chemical mechanisms, typical product characteristics, advantages and limitations, and their implications for large‐scale production and device integration. We begin with classical solid‐state methods, still widely employed due to their simplicity and robustness, before addressing wet‐chemical, gas‐phase, and high‐throughput techniques that offer greater flexibility and speed in materials discovery.

## 2 Solid‐State and Mechanochemical Synthesis

Solid‐state and mechanochemical synthesis routes represent two of the most established and scalable methods for the fabrication of HEMs. They are widely used for laboratory‐ and pilot‐scale production due to their robustness, low precursor cost, and wide compositional applicability. Despite these advantages, both methods pose unique challenges when targeting single‐phase, nanostructured, and application‐specific materials, a key requirement for functional high‐entropy systems.

Conventional solid‐state synthesis typically involves two sequential steps: intimate precursor mixing followed by high‐temperature calcination to induce crystallization and phase formation. This process benefits from its simplicity and scalability yet is fundamentally limited by its reliance on solid‐state diffusion (on the order of 10^−15^ m^2^ s^−1^), which is several orders of magnitude slower than in solution. As a result, long reaction times and high temperatures, often exceeding 1000 °C, are necessary to drive the reaction to completion. These conditions favor the formation of large, irregularly shaped grains with low surface area, which are often unsuitable for catalysis or interfacial electrochemical processes where high active surface areas are desired.^[^ ^26^ ^]^

**Figure 2:**
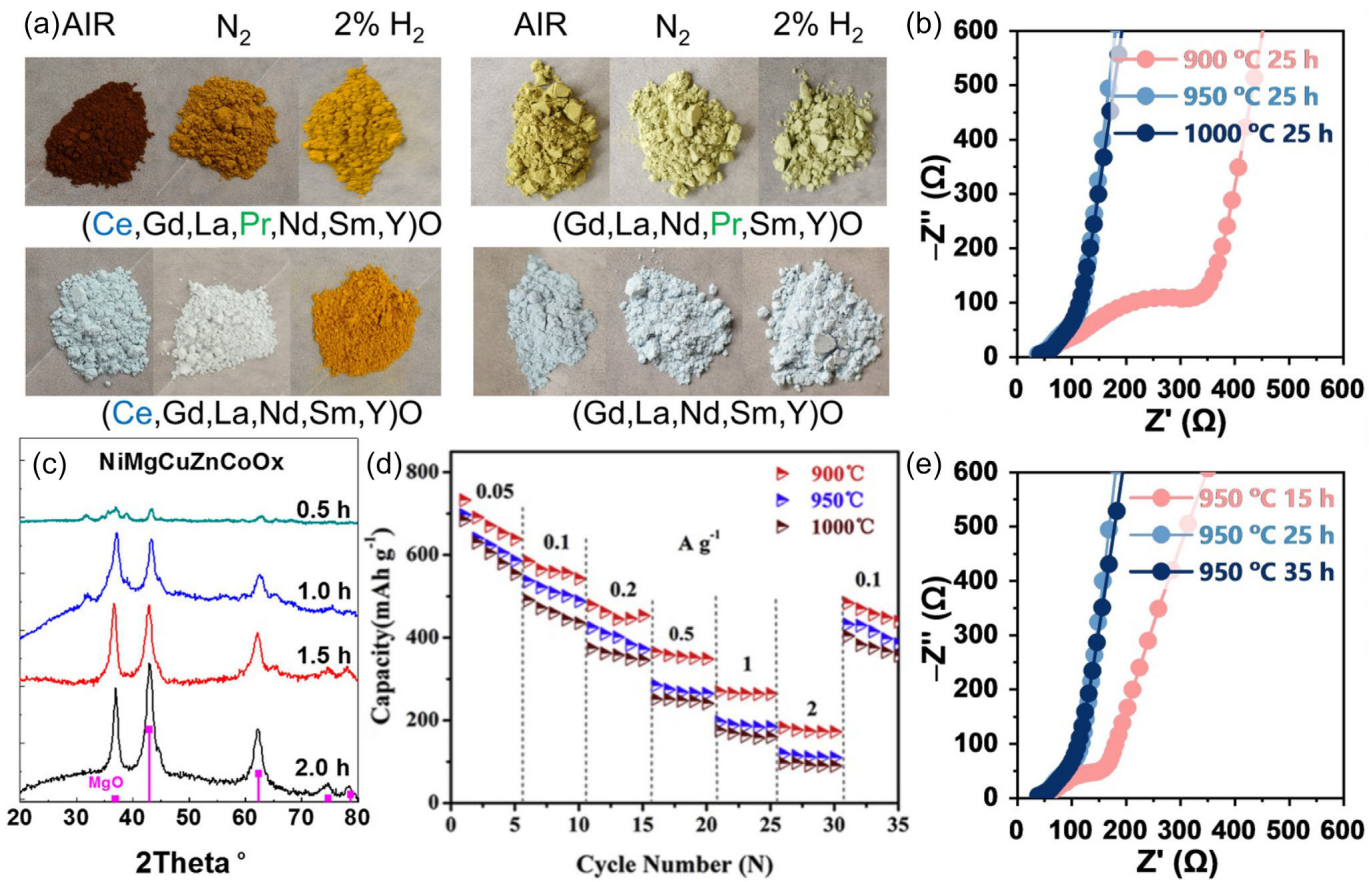
a) Photographs of ground sintered samples comparing the change in color with composition and sintering atmosphere. Reproduced with permission.^[^ ^29^ ^]^ Copyright 2020, John Wiley and Sons. b) Nyquist plots of Na_4.9_Sm_0.3_Y_0.2_Gd_0.2_La_0.1_Al_0.1_Zr_0.1_Si_4_O_12_ obtained at different sintering temperatures. Reproduced with permission.^[^ ^33^ ^]^ Copyright 2023, John Wiley and Sons. c) XRD patterns for (NiMgCuZnCo)O_x_ synthesized by ball milling for different times. Reproduced with permission.^[^ ^34^ ^]^ Copyright 2019, American Chemical Society. d) Rate capabilities of (FeCoNiCrMn)_3_O_4_ sintered at varying temperatures from 0.05 to 2 A g^−1^. Reproduced with permission.^[^ ^35^ ^]^ Copyright 2020, Elsevier. e) Nyquist plots of Na_4.9_Sm_0.3_Y_0.2_Gd_0.2_La_0.1_Al_0.1_Zr_0.1_Si_4_O_12_ obtained at different sintering times. Reproduced with permission.^[^ ^33^ ^]^ Copyright 2023, John Wiley and Sons.

Synthesis outcomes in solid‐state reactions are highly sensitive to parameters such as precursor choice, particle size, calcination temperature and duration, heating/cooling rates, and, critically, the synthesis atmosphere. Tailoring these variables can lead to dramatically different outcomes in terms of crystallinity, phase composition, and defect formation. For instance, adjusting the oxygen partial pressure [*p*(O_2_)] during calcination influences the formation of oxygen vacancies or interstitials, which in turn affect the electrical conductivity, redox behavior, or catalytic activity of the resulting material.^[^ ^27^, ^28^ ^]^ Moreover, the stability of multivalent cations such as Mn, Fe, or Pr is highly atmosphere‐dependent: Pr‐based high‐entropy oxides may crystallise in cubic symmetry under oxidizing conditions, but transition to monoclinic phases under inert or reducing environments.^[^ ^29^, ^30^, ^31^ ^]^ Faster quenching conditions can also help in incorporating ions with a larger size mismatch, like Ca^2+^ in MgCoNiCuZnO, in near equimolar conditions.^[^ ^32^ ^]^ These effects are exemplified in **Figure** **2**, which demonstrates how sintering temperature and atmosphere affect the composition, microstructure, and electrochemical performance of various HEMs.

Solid‐state synthesis is particularly challenged when incorporating volatile species like Li or Na, which readily evaporate at elevated temperatures. This issue is commonly addressed by using excess precursor amounts, although this complicates stoichiometric control and can lead to phase impurities.^[^ ^36^, ^37^, ^38^, ^39^, ^40^ ^]^ To overcome these kinetic and thermal limitations, mechanochemical synthesis, particularly high‐energy ball milling, has emerged as a powerful alternative. In this method, the mechanical energy imparted by the collision of grinding media causes particle size reduction, localized heating, and enhanced atomic diffusion. These effects enable the formation of nanocrystalline or even amorphous single‐phase materials, often without requiring a subsequent thermal treatment.^[^ ^34^, ^41^ ^]^ The key advantages of mechanochemical synthesis include low processing temperatures, rapid processing times, and the ability to synthesize metastable or non‐equilibrium phases, including fluorides, sulfides, and mixed halide HEMs.^[^ ^42^, ^43^, ^44^, ^45^, ^46^ ^]^

However, the method comes with drawbacks. Mechanochemical routes are energy‐intensive, and controlling parameters such as milling time, ball‐to‐powder ratio, and media hardness is critical to avoid contamination, excessive amorphization, or formation of undesired secondary phases. The resulting nanocrystalline products often exhibit high defect densities, which may enhance certain properties (e.g., catalytic activity), but can be detrimental for others (e.g., ionic conductivity or long‐term stability). Post‐synthetic annealing is frequently employed to reduce surface defects or tune the electronic environment. Furthermore, hybrid methods, such as reactive grinding with alkali halide salts followed by leaching, can introduce beneficial porosity for catalytic applications, although this remains underexplored for many HEM systems.^[^ ^47^ ^]^

Mechanochemical synthesis is particularly attractive for non‐oxidic high‐entropy systems, such as sulfides or fluorides, where solution‐based synthesis routes are limited due to solubility or reactivity constraints. For example, S_8_, MF_x_, or mixed halide precursors have been used successfully to generate complex anionic HEM structures.^[^ ^42^, ^43^, ^44^, ^45^ ^]^

A variety of high‐entropy ceramics, ranging from spinels and perovskites to fluorite and layered structures, have been synthesized using solid‐state and mechanochemical techniques. Some studies, such as those by Chen et al. and Gao et al., have demonstrated significant functional performance using such materials for catalytic CO_2_ conversion and CO oxidation.^[^ ^34^, ^48^ ^]^ Others have extended the approach to novel classes such as high‐entropy oxyfluorides and carbon‐supported catalysts.^[^ ^41^, ^44^ ^]^ An overview of representative materials and their key properties is provided in **Table** **1**.

**Table 1:** Overview of HEOs and properties prepared by solid‐state synthesis.

Formula	Structure	Application	Properties	Refs.
(CuNiFeCoZnMnMg)F_2_	Fluorite	Catalysis: Oxygen evolution reaction (OER)	Overpotential of 292 mV at 10 mA cm^−2^; Tafel slope of 39 mV dec^−1^	[44]
La(CoFeNiMnV)O_3_	Perovskite	Catalysis: CO oxidation	Stability over ≈350 h; Thermal stability at >500 °C Catalytic performance improved due to higher specific surface area	[48]
CuCeO*_x_*‐(NiMgCuZnCo)O*_x_*	Composite heterostructure	Catalysis: CO oxidation	High thermal stability	[49]
A_32_Ti_8_Sn_8_Nb_4_Ta_4_Me_8_O_96_ (A = Ba, Sr; Me = Fe, Ga)	Perovskite	Catalysis: Photocatalysis	Cr(VI) reduction under UV light	[50]
NaNi_0.12_Cu_0.12_Mg_0.12_Fe_0.15_Co_0.15_Mn_0.1_Ti_0.1_Sn_0.1_Sb_0.04_O_2_	Layered (O_3_‐type)	Energy storage: Na‐ion cathode	Capacity retention of ≈83% after 500 cycles	[51]
Mg_0.2_Co_0.2_Ni_0.2_Cu_0.2_Zn_0.2_O	Rock‐salt	Energy storage: Li‐ion anode	Specific capacity of 920 mAh g^−1^ after 300 cycles	[52]
Na_0.83_Li_0.1_Ni_0.25_Co_0.2_Mn_0.15_Ti_0.15_Sn_0.15_O_2−_ *_δ_*	Layered (O_3_‐type)	Energy storage: Na‐ion cathode	77% capacity retention at 10C; 87% capacity retention after 200 cycles; Specific capacity of 109 mAh g^−1^; Good full‐cell performance, with 84% capacity retention after 100 cycles	[53]
(Ba_0.8_Bi_0.2_)(Ti_0.58_Zr_0.14_Sc_0.18_Mg_0.04_Nb_0.05_Ta_0.01_)O_3_ (Ba_0.8_Bi_0.2_)(Ti_0.58_Zr_0.14_Sc_0.18_Zn_0.04_Nb_0.05_Ta_0.01_)O_3_	Perovskite	Energy storage: Capacitor (dielectric)	Energy density of 5.18 J cm^−3^; ≈94% efficiency at 640 kV cm^−1^; Smaller grain sizes improve energy storage performance	[54]
Sr(TiZrHfSnRu)O_3_	Perovskite	Catalysis: Nitrogen oxidation	Yield rate of 39.0 µmol mg^−1^ h^−1^; Faradaic efficiency of ≈33%; Higher concentration of oxygen vacancies reduces energy barrier for N_2_ oxidation	[55]

### 2.1 Functional Implications of Solid‐State and Mechanochemical Synthesis

**Figure 3:**
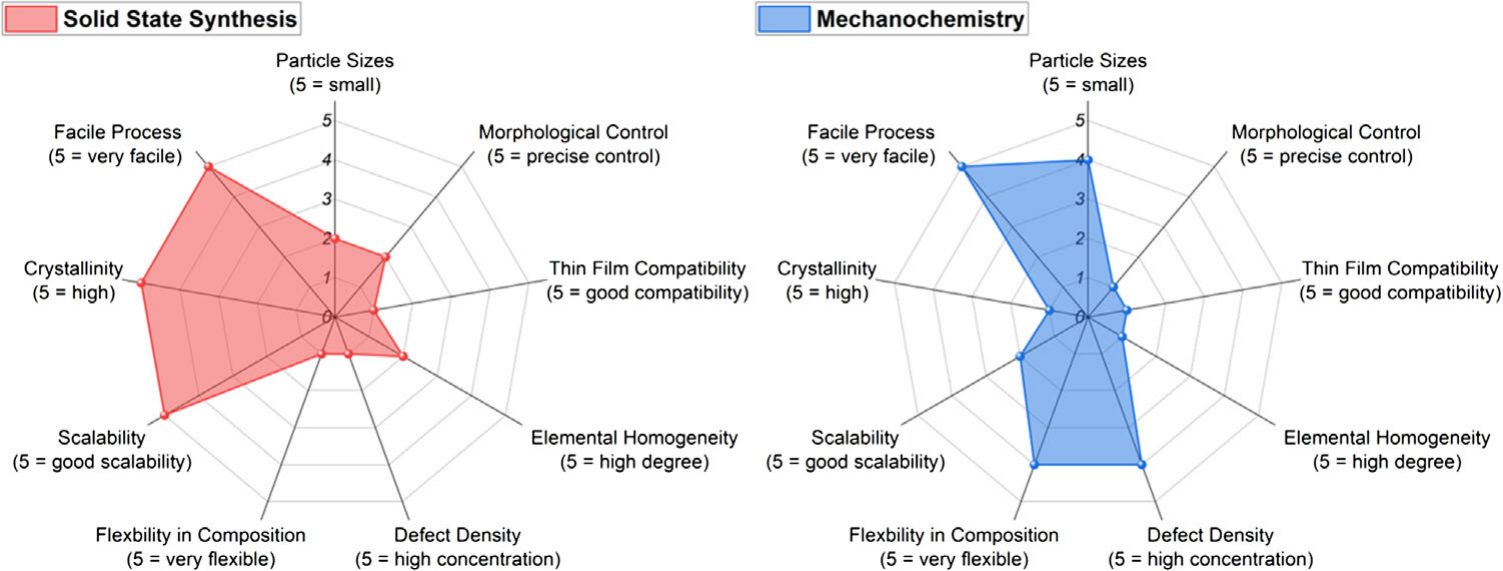
Radar charts for solid‐state and mechanochemical synthesis methods.

Solid‐state and mechanochemical synthesis routes represent two of the most established and scalable approaches for the fabrication of HEMs. Their widespread use stems from the compositional flexibility, robustness, and cost‐effectiveness. However, the material characteristics differ substantially, especially in terms of particle morphology, crystallinity, defect structure, and scalability. These features directly affect the suitability of the resulting materials for targeted applications. Below, we analyze both synthesis approaches with respect to the key functional metrics defined in the radar charts presented in **Figure** **3**.

#### 2.1.1 Particle Size

Mechanochemical synthesis enables significant particle size reduction through repeated fracturing and cold‐welding during high‐energy ball milling. This typically yields nanoparticles or submicron grains with high specific surface areas, which are particularly desirable in catalysis and/or electrochemical energy storage. In contrast, solid‐state synthesis, which involves prolonged high‐temperature treatments (>1000 °C), produces coarse‐grained particles with sizes often in the range of several micrometers unless intermediate grinding steps are introduced. These larger particles can be a disadvantage in surface‐sensitive applications but may offer higher thermal and mechanical stability for bulk ceramics.

#### 2.1.2 Morphological Control

Both techniques offer only limited morphological control. In solid‐state synthesis, particle shape and agglomeration are governed largely by sintering temperature, precursor reactivity, and atmosphere. Morphologies tend to be irregular and dense, as shown in various scanning electron micrographs across the literature. Mechanochemical synthesis generally results in agglomerated, irregular particles with rough surfaces and poorly defined shapes. While some degree of porosity or nanostructuring can be achieved, e.g., through salt‐assisted grinding and post‐leaching steps, precise tailoring of morphologies (e.g., formation of hollow spheres, rods, or platelets) remains challenging without hybrid strategies.

#### 2.1.3 Thin Film Compatibility

Neither technique is inherently tailored for thin‐film fabrication. Solid‐state synthesis yields coarse particles that require further processing, such as milling, dispersion, or slurry preparation, for deposition techniques like screen printing or tape casting. Mechanochemical synthesis offers finer powders that are more dispersible and potentially useful in colloidal or ink‐based methods, but their poor film‐forming behavior (e.g., cracking, pinhole formation) often necessitates additional binder or annealing steps. Thin‐film integration thus remains a bottleneck for both routes, especially compared to solution‐based methods.

#### 2.1.4 Elemental Homogeneity

Mechanochemical synthesis offers good elemental mixing due to the repeated mechanical deformation and intimate contact between different reactants, even in diffusion‐limited systems. This makes it especially suitable for systems with sluggish diffusion, such as rare‐earth‐rich oxides or complex fluorides. Solid‐state synthesis can also yield uniform phases, but this typically requires extended calcination times and thorough precursor mixing. Incomplete reaction or sintering gradients can lead to phase segregation or inhomogeneous dopant distributions, particularly problematic for multi‐cation systems with volatile or redox‐sensitive elements (e.g., Mn, Li).

#### 2.1.5 Defect Density

Mechanochemical routes inherently introduce a high concentration of structural defects, including vacancies, dislocations, and grain boundaries. These defects can serve as active sites in catalysis or facilitate ion migration in certain applications. However, excessive disorder may impede electronic transport, reduce structural stability, or promote unwanted side reactions. In contrast, solid‐state synthesis allows for better control over defect concentrations through careful adjustment of calcination temperature, time, and *p*(O_2_). For example, modifying the oxygen partial pressure during sintering can promote the controlled formation of oxygen vacancies or stabilize specific valence states in multivalent cations.

#### 2.1.6 Flexibility in Composition

Both synthesis techniques allow for broad compositional tailoring, including equimolar and non‐equimolar mixtures of transition metals, rare earths, and main‐group elements. Mechanochemical methods are especially advantageous for synthesizing non‐oxide HEMs, such as fluorides, sulfides, or halides, which are difficult to access via wet‐chemical approaches due to solubility or hydrolysis issues. Examples include the successful use of S_8_ and MF*_x_* precursors to obtain multi‐anionic systems. Solid‐state synthesis, while compatible with a wide variety of oxide compositions, can suffer from the loss of volatile elements like Li or Na during high‐temperature steps, necessitating excess precursor or rapid heating strategies.

#### 2.1.7 Scalability

Solid‐state synthesis is highly scalable and routinely employed in the ceramic and battery industries. Its reliance on simple furnaces, low‐cost precursors, and batch processing makes it attractive for upscaling, particularly for bulk materials. Mechanochemical synthesis is also scalable, especially with the advent of industrial planetary and attritor mills, but still faces challenges related to contamination from milling media, reproducibility, and energy efficiency. These limitations are especially critical when working with reactive or phase‐sensitive systems.

#### 2.1.8 Crystallinity

Solid‐state synthesis generally yields highly crystalline products due to the elevated temperatures and long reaction times that promote complete phase formation and grain growth. This is advantageous in applications requiring long‐range order or phase purity, such as in dielectric or electrolyte materials. Conversely, mechanochemical synthesis often produces nanocrystalline or even amorphous phases, particularly when ball milling is performed at room temperature without post‐annealing. While this can be advantageous for catalysis or surface‐dominated reactions, it may compromise properties like ionic conductivity or phase stability.

#### 2.1.9 Process Simplicity

Process simplicity is a decisive factor when selecting a synthesis route, especially for high‐throughput screening, rapid prototyping, or industrial implementation. Solid‐state synthesis is widely regarded as one of the most straightforward methods for preparing HEMs, requiring only basic equipment, minimal chemical handling, and relatively few processing steps. The direct mixing and thermal treatment of oxide precursors eliminates the need for solvents, complex ligands, or stringent reaction atmospheres, which significantly lowers the barrier for reproducibility and scale‐up. Mechanochemical synthesis also benefits from operational simplicity. Specifically, it avoids high‐temperature steps and liquid reagents, relying instead on mechanical energy to drive solid‐state reactions. However, careful control over milling parameters (e.g., time, speed, atmosphere, and ball‐to‐powder ratio) is required to ensure consistent product quality. Despite this, both methods offer robust, solvent‐free pathways that stand out in terms of simplicity compared to multistep wet‐chemical processes, particularly when the goal is to quickly access a wide compositional space.

To clearly visualize the implications of these synthesis‐induced properties on functionality, we introduce radar charts that map each synthesis route (e.g., solid‐state, mechanochemical) against this set of performance‐relevant descriptors (Figure 3). These charts will be presented at the end of each synthesis section and act as a conceptual bridge to functional applications. They allow for rapid comparison of synthesis methods in terms of their suitability for electronic, catalytic, electrochemical, or sensor applications and support the development of decision frameworks for researchers working on application‐driven HEM development.

## 3 Solution‐Based Synthesis Methods

Solution‐based synthesis methods, including co‐precipitation, sol‐gel, solution combustion, and solvothermal techniques, are extensively utilized for the fabrication of high‐entropy ceramics due to their ability to provide precise control over particle size, morphology, and precursor homogeneity (representative materials and their key properties are provided in **Table** **2**). These wet‐chemical approaches operate via bottom‐up assembly of atomic or molecular species into nanoscale structures, offering modularity, scalability, and tunability that are essential for tailoring functional properties in multicomponent systems.^[^ ^56^, ^57^ ^]^ The kinetic and thermodynamic parameters inherent to these processes, such as precursor chemistry, reaction temperature, heating and cooling rates, reagent concentration, mixing dynamics, ligand interactions, and solvent characteristics, critically influence nucleation and growth mechanisms, thereby governing particle size distribution, crystallinity, and compositional uniformity. Consequently, a comprehensive understanding of these variables is paramount for reproducible and controlled synthesis of HEMs.

**Table 2:** Overview of HEMs [HEOs and Prussian blue/white analogues (PBA/PWAs)] and properties prepared using co‐precipitation synthesis.

Formula	Structure	Application	Properties	Refs.
(Co_0.2_Cr_0.2_Fe_0.2_Mn_0.2_Ni_0.2_)_3_O_4_	Spinel	Catalysis: OER	Overpotential of 220 mV at 10 mA cm^−2^; Current density of ≈335 mA cm^−2^	[92]
(Co_0.2_Cr_0.2_Fe_0.2_Mn_0.2_Ni_0.2_)_3_O_4_	Spinel	Catalysis: Propane conversion	90% propane conversion at 325 °C; Using NaOH as precipitant gives the most oxygen vacancies	[83]
La(Co_0.33_Cr_0.16_Fe_0.16_Ni_0.16_Mn_0.16_)O_3_	Perovskite	Catalysis: OER	Overpotential of 325 mV at 10 mA cm^−2^; Stability over 50 h	[60]
(Al_0.2_Co_0.2_Cr_0.2_Mn_0.2_Ni_0.2_)_3_O_4_	Spinel	Energy storage: “Battery‐like” supercapacitor	Specific surface area of 90 m^2^ g^−1^ at 550 °C; Specific capacity of 318 mAh g^−1^ at 1 A g^−1^; Higher specific surface area improves the electrode performance	[57]
(Co_0.2_Cr_0.2_Fe_0.2_Mn_0.2_Ni_0.2_)_3_O_4_	Spinel	Energy storage: Supercapacitor	Capacitance of 239 F g^−1^; Energy density of 24.1 Wh kg^−1^ at 0.5 A g^−1^; Capacitance retention of 76% after 1000 cycles	[93]
La(CoCrFeMnNiAl_0.5_)_1/5.5_O_3_	Perovskite	Energy storage: Supercapacitor	Specific capacitance of 354 F g^−1^ at 1 A g^−1^; Capacitance retention of 89% after 2000 cycles; Al^3+^‐doped electrodes have lower diffusion impedance and faster ion diffusion	[62]
LiNi_0.8_Mn_0.13_Ti_0.02_Mg_0.02_Nb_0.01_Mo_0.02_O_2_	Layered (O_3_‐type)	Energy storage: Li‐ion cathode	Volumetric strain of 0.3%	[63]
Co_0.2_Cu_0.2_Mg_0.2_Ni_0.2_Zn_0.2_O	Rock‐salt	Catalysis: CO oxidation	High thermal stability	[95]
(Gd_0.2_Nd_0.2_La_0.2_Sm_0.2_Y_0.2_)CoO_3_	Perovskite	Metal oxide semiconductor	Medium to low band gap on increasing temperature; Slow quenching results in multi‐phase mixture	[64]
Gd_0.2_La_0.2_Ce_0.2_Hf_0.2_Zr_0.2_O_2_	Fluorite	Catalysis: Photocatalysis	Reduction of Cr(VI)	[65]
K_0.38_(Co_0.14_Cu_0.29_Mn_0.14_Ni_0.14_Zn_0.29_)[Fe(CN)_6_]_0.62□0.38_·4.11H_2_O	PBA	Supercapacitor, OER	Specific capacity of 45 mAh g^−1^ at 1 A g^−1^ and high cycling stability; 242 mV at 10 mA cm^−2^ and stability over 60 h	[102]
Li_0.16_Mg_0.16_Co_0.16_Mn_0.16_Cu_0.16_Zn_0.16_O	Rock‐salt	Energy storage: Solid electrolyte	Ionic conductivity of ≈10^−3^ S cm^−1^	[25]
(Cr_0.2_Mn_0.2_Fe_0.2_Ni_0.2_Zn_0.2_)_3_O_4_	Spinel	Catalysis: OER	Overpotential of 295 mV at 10 mA cm^−2^; Tafel slope of 53.7 mV dec^−1^	[66]
Na_1.46_Mn_0.3_Co_0.3_Fe_0.133_Ni_0.133_Cu_0.133_[Fe(CN)_6_]_0.86□0.14_·1.74H_2_O	PBA	Energy storage: Na‐ion cathode	Specific capacity of 60 mAh g^−1^ after 10 000 cycles at 800 mA g^−1^;	[67]
(Na_1.65_Mn_0.4_Fe_0.12_Ni_0.12_Cu_0.12_Co_0.12_Cd_0.12_[Fe(CN)_6_]_0.92□0.08_⋅1.09H_2_O)	PWA	Energy storage: Na‐ion cathode	Improved tolerance for lattice expansion and shrinkage	[68]

Co‐precipitation synthesis is a straightforward and commonly employed method for producing oxide nanoparticles, often used interchangeably with reverse co‐precipitation, where the sequence of steps is reversed. The primary goal is to create multicomponent materials by facilitating the formation of intermediate precipitates.^[^ ^58^ ^]^ Typically, co‐precipitation involves combining aqueous metal salts with a base at elevated temperatures, leading to precipitation.^[^ ^59^ ^]^ This approach fosters a well‐integrated mixture of components during precipitation, maintaining chemical homogeneity through calcination and enabling control over multicomponent oxide precipitation.

Beyond co‐precipitation, sol‐gel synthesis constitutes a powerful alternative, leveraging hydrolysis and polycondensation reactions of metal alkoxides or salts to form homogeneous gels that can be subsequently calcined into highly uniform oxides. The sol‐gel process allows molecular‐level mixing of multiple metal species, affording exceptional compositional control and the potential to engineer porosity, surface area, and particle morphology through manipulation of gelation kinetics, solvent environment, and drying conditions. This method has been applied successfully to synthesize a broad range of high‐entropy oxides with controlled nanostructures suitable for catalytic and electrochemical applications.^[^ ^60^, ^61^, ^62^, ^63^ ^]^ The ability to incorporate organic ligands and templating agents in sol‐gel routes further facilitates the design of hierarchical porosity and tailored surface chemistries.

Solution combustion synthesis (SCS) represents another wet‐chemical approach distinguished by its rapid and exothermic reaction, whereby a fuel (e.g., glycine, urea) combusts with oxidizing metal nitrates, producing nanoparticulate oxides in a self‐sustained reaction. SCS offers advantages such as short synthesis times, low energy input, and the formation of porous, high‐surface‐area materials. However, controlling phase purity and particle size can be challenging due to the highly energetic nature of the process, which may induce rapid nucleation and uncontrolled growth.^[^ ^64^, ^65^, ^66^ ^]^ Recent adaptations of SCS to HEMs have demonstrated promising catalytic and electrochemical performances, though further optimization is necessary to improve uniformity and scalability.

Solvothermal synthesis exploits elevated temperatures and pressures in sealed reactors to facilitate the crystallization of complex oxides from solution, enabling precise control over particle size, morphology, and crystallinity. This method is particularly effective for synthesizing metastable or complex phases that are difficult to access via conventional solid‐state routes. The tunability of solvent composition, reaction temperature, pressure, and time allows fine adjustment of nucleation and growth kinetics, resulting in well‐defined nanostructures with tailored functional properties.^[^ ^66^, ^67^, ^68^, ^69^ ^]^ Solvothermal approaches have been extended to the synthesis of high‐entropy oxides and other ceramic systems, where the high‐pressure environment enhances diffusion and mixing at the atomic scale, promoting phase homogeneity.

Electrospinning is a specialized solution‐based processing technique that enables the fabrication of continuous nanofibers with high surface area‐to‐volume ratios. When a precursor solution containing multiple metal cations is electrospun, the ions are co‐distributed within a polymer matrix and drawn into nanofibrous structures under the influence of an electrostatic field. Subsequent thermal treatment (e.g., calcination) removes the organic components and leads to the formation of oxide fibers with uniform elemental distribution, making the method well‐suited for the synthesis of high‐entropy materials. The morphology of the resulting particles, typically fibrous or porous, is directly influenced by the electrospinning parameters (e.g., voltage, flow rate, and solvent system) and the polymer‐to‐metal ratio, allowing for tailored control over fiber diameter and surface texture. This morphological control is particularly advantageous for applications where anisotropic architectures or hierarchical porosity enhance functional performance, such as in sensing, catalysis, or energy storage.^[^ ^70^, ^71^, ^72^, ^73^, ^74^, ^75^ ^]^

Despite the clear advantages of these solution‐based methods in terms of control and versatility, challenges remain regarding their scalability and reproducibility. Conventional batch syntheses suffer from limitations in mixing efficiency and heat transfer, leading to batch‐to‐batch variability. Continuous flow reactors have emerged as a promising solution, enabling enhanced mass and heat transport due to small reactor volumes and high surface‐to‐volume ratios. Flow synthesis permits automation and fine control over reaction parameters such as residence time, temperature, and reagent feed rates, which is crucial for reproducible large‐scale production of high‐entropy nanoparticles without compromising compositional fidelity.^[^ ^76^ ^]^ The modular nature of flow setups further allows integration of inline characterization tools, accelerating process optimization and materials discovery.

In summary, solution‐based synthesis techniques offer a multifaceted toolkit for engineering HEMs with finely tuned structural and functional attributes. Co‐precipitation and reverse co‐precipitation remain cornerstone methods for producing homogeneous precursors and controlling particle size, while sol‐gel, solution combustion, and solvothermal routes extend capabilities towards hierarchical porosity, rapid synthesis, and complex phase formation. Advances in continuous flow synthesis promise to bridge the gap between laboratory‐scale experimentation and industrial‐scale production, facilitating the broader application of high‐entropy ceramics in catalysis, energy storage, and electronic devices. The selection and optimization of these synthesis strategies must carefully balance parameters influencing phase purity, morphology, defect concentration, and scalability to harness the full potential of HEMs in emerging technologies.

### 3.1 Co‐Precipitation and Reverse Co‐Precipitation

**Figure 4:**
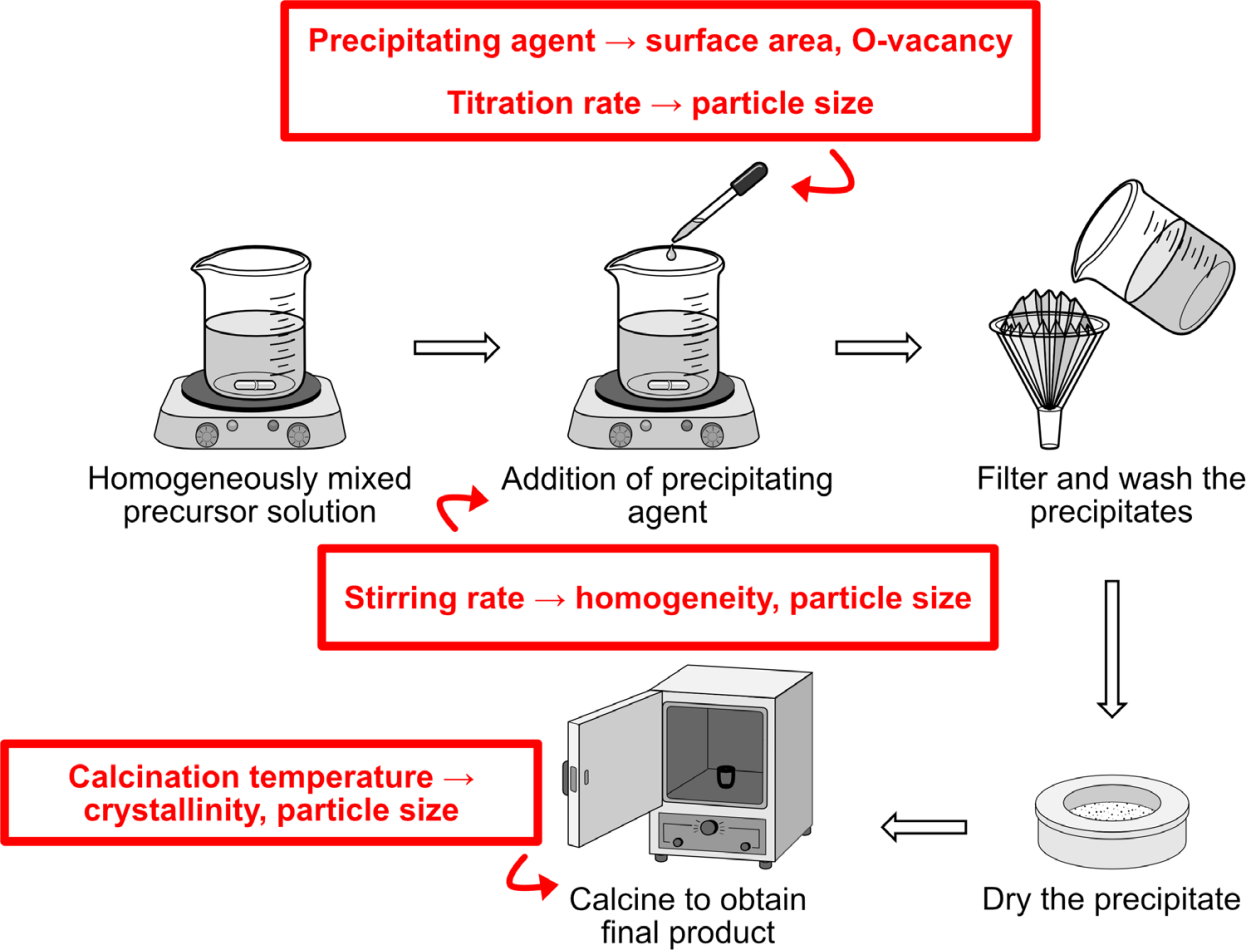
Schematic illustration of the co‐precipitation synthesis route, highlighting key factors affecting material properties.

Among these methods, co‐precipitation and its variant reverse co‐precipitation are widely employed due to their operational simplicity and effectiveness in producing multicomponent oxide nanoparticles with intimate elemental mixing at early synthesis stages. In typical co‐precipitation, an acidic aqueous solution containing metal salt precursors is slowly introduced into a large volume of basic solution, facilitating precipitation by pH stabilization. Conversely, reverse co‐precipitation involves the gradual addition of a basic solution into the metal precursor mixture, resulting in simultaneous precipitation of multiple cations under a nearly constant pH environment (illustrated in **Figure** **4**). This subtle procedural difference significantly impacts phase homogeneity and stoichiometric retention, with reverse co‐precipitation often yielding more uniform multicomponent hydroxide or carbonate intermediates that, upon calcination, transform into single‐phase oxides with reduced impurity formation.^[^ ^58^, ^59^, ^77^, ^78^, ^79^, ^80^ ^]^

Co‐precipitation methods offer considerable versatility in tuning particle size and morphology through control of reaction parameters such as temperature, stirring rate, and the nature of precipitating agents.^[^ ^81^ ^]^ Different precursor systems (e.g., oxalates, hydroxides, or carbonates) influence material properties and can be selected by adjusting the precipitant agent.^[^ ^82^ ^]^ For example, the choice between NaOH and Na_2_CO_3_ as precipitants influences not only particle size but also defect chemistry; NaOH can promote oxygen vacancy formation beneficial for catalytic activity, whereas Na_2_CO_3_ decomposition releases CO_2_, increasing mesoporosity and specific surface area.^[^ ^83^ ^]^ Ngoepe et al. conducted an in‐depth study on how process parameters such as pH, reaction temperature, reaction time, and stirring speed impact the precipitation of carbonate precursors.^[^ ^84^ ^]^

The pH critically affects the precipitation of individual cations, exemplified by the requirement of alkaline conditions (pH > 10) for Mg(OH)_2_ formation to avoid deviations from target stoichiometry.^[^ ^58^, ^85^ ^]^ The incorporation of capping agents such as ethylene glycol, glycerol, or oleic acid further refines particle growth, restricting agglomeration and enabling fine control over crystallite dimensions and lattice strain, which in turn influence electronic band structures.^[^ ^86^, ^87^, ^88^ ^]^ Intermediate sonication‐assisted peptization steps have demonstrated reductions in agglomerate size and enhanced colloidal stability, exemplified by particle sizes below 3 nm in fluorite‐type high‐entropy oxides.^[^ ^89^, ^90^ ^]^ Similar ultrasonication steps have also been used to successfully prepare sub 3 nm sized HEA particles with a uniform pore distribution, making them attractive for use in catalysis.^[^ ^91^ ^]^ In an acidic medium, the growth of hydroxide precipitates is inhibited, and sonication facilitates the breakdown of larger agglomerates. Protonation of hydroxyl groups allows nitrate anions to form a Stern layer with positively charged particles, stabilizing the sol.^[^ ^90^ ^]^ Co‐precipitation can also produce unique structures, such as spinel‐type spherical or quasi‐spherical particles, with sizes ranging from 20 to 60 nm.^[^ ^92^, ^93^, ^94^ ^]^ These particles typically exhibit higher specific surface areas and lower oxygen‐vacancy concentrations than those synthesized via sol‐gel methods. For instance, Chen et al. reported that (NiCoMgZnCu)O prepared by co‐precipitation has a Brunauer‐Emmett‐Teller (BET) surface area of 28 m^2^ g^−1^, compared to 2 m^2^ g^−1^ from ball milling.^[^ ^95^ ^]^ Thus, co‐precipitation is an ideal route for preparing materials for catalytic applications, allowing various methods to control the specific surface area of the synthesized particles. Slow dropwise addition of the precipitating solution is also crucial to obtaining nanoparticles with a uniform size. HEMs can also be engineered to introduce lattice strain in their structures, which can further help in oxygen diffusion through the lattice, increasing the OER activity of prepared HEOs.^[^ ^96^ ^]^ Epitaxial coatings, which can be used to improve the structural stability of electrodes in Li‐ion batteries while maintaining fast reaction kinetics, can also be developed with co‐precipitation routes. This opens up another facet of surface engineering available for the improvement of device characteristics.^[^ ^97^ ^]^

This technique can also be used to synthesize complex structures like metal‐organic frameworks (MOFs).^[^ ^98^ ^]^ If all precursor elements precipitate at a similar pH, forward co‐precipitation may also be effective, as demonstrated by Spiridigliozzi et al.^[^ ^99^ ^]^ for fluorite rare‐earth oxides and Usharani et al.^[^ ^100^ ^]^ for rock‐salt transition‐metal oxides. However, co‐precipitation methods require extensive washing, drying, and calcination to achieve high‐purity crystalline phases.^[^ ^101^ ^]^ In comparing forward and reverse co‐precipitations, crystallite sizes are generally smaller in the forward reaction, suggesting a higher band gap due to decreased particle size and increased confinement of electrons and holes. However, Albadi et al. found that samples from forward co‐precipitation have larger particle sizes despite smaller crystallites, leading to a smaller band gap.^[^ ^80^ ^]^ This phenomenon may result from a stronger aggregation of crystallites in the forward reaction compared to reverse co‐precipitation samples.

Spiridigliozzi et al. also developed a predictor for designing HEOs based on rare‐earth oxides, which typically form fluorite, bixbyite, or biphasic structures. The classification can be predicted by assessing the standard deviation of the cationic radii of the system, namely deviations above 0.095 yield a single‐phase fluorite structure, while those below 0.095 indicate a transition to bixbyite structure at higher annealing temperatures.^[^ ^99^ ^]^ Kumbhakar et al. confirmed this classification in their high‐throughput, co‐precipitation synthesis of high‐entropy rare‐earth oxides.^[^ ^30^ ^]^

### 3.2 Microwave‐Assisted Synthesis

Microwave‐assisted synthesis techniques have emerged as highly effective approaches for the rapid and energy‐efficient fabrication of high‐purity nanoscale powders, dramatically reducing reaction times from several hours to minutes. This method facilitates low‐temperature processing through direct volumetric heating by microwave radiation, resulting in efficient energy transfer, reduced thermal gradients, and enhanced environmental sustainability compared to conventional thermal routes.^[^ ^69^, ^103^, ^104^, ^105^ ^]^ Due to these advantages, microwave synthesis is particularly well‐suited for producing nanostructured high‐entropy oxides (HEOs) with controlled composition and morphology.

This technique has been successfully applied to synthesize a variety of HEO systems, including rock‐salt structured Co_0.2_Cu_0.2_Mg_0.2_Ni_0.2_Zn_0.2_O^[^ ^103^, ^106^ ^]^ as well as several spinel‐type compounds such as (Al_0.2_Co_0.2_Fe_0.2_Mn_0.2_Ni_0.2_)_3_O_4_,^[^ ^107^ ^]^ (Co_0.2_Cr_0.2_Fe_0.2_Mn_0.2_Ni_0.2_)_3_O_4_,^[^ ^108^, ^109^ ^]^ and doped variants like (Cr_0.2_Co_0.2‐_ *_x_* Fe_0.2_Mn_0.2_Ni_0.2_Zn*_x_*)_3_O_4_,^[^ ^109^ ^]^ as detailed in **Table** **3**. A related method, photonic curing, employs intense radiative heating from light sources to synthesize nanostructured HEOs from thermally sensitive precursors, thus broadening the scope of low‐temperature fabrication techniques.^[^ ^110^ ^]^

**Table 3:** Overview of HEOs and properties prepared by microwave‐assisted synthesis.

Formula	Structure	Application	Properties	Refs.
Co_0.2_Cu_0.2_Mg_0.2_Ni_0.2_Zn_0.2_O	Rock‐salt	Energy storage: Li‐ion anode	Specific capacity of >250 mA g^−1^ at 5 A g^−1^; Capacity retention of ≈98% after 1000 cycles at 1 A g^−1^; Uniform size distribution of nanoparticles	[103]
(FeCoNi_2_CrMn)_3_O_4_	Spinel	Catalysis: OER	Overpotential of 260 mV at 10 mA cm^−2^; Tafel slope of 50.1 mV dec^−1^; Increasing metal valence state improves catalytic activity	[115]
Co_0.2_Cu_0.2_Mg_0.2_Ni_0.2_Zn_0.2_O	Rock‐salt	Energy storage: Cathode coating for Li‒S batteries	Coulomb efficiency of ≈99% over 500 cycles at C/5	[106]
(FeMnNiV)O/MXene	Composite	Catalysis: OER	Overpotential of 331 mV at 10 mA cm^−2^; Tafel slope of 71 mV dec^−1^	[123]
(Co_0.2_Cr_0.2_Mg_0.2_Mn_0.2_Fe_0.2_)_3_O_4_	Spinel	Energy storage: Li‐ion anode	Specific capacity of >1000 mAh g^−1^ at 0.1 A g^−1^	[124]
(NiCoMnCuZnMg)Fe_2_O_4_ (NiCoMnCuZnMg)FeCoO_4_	Spinel	Catalysis: OER	Overpotential of 460 mV at 10 mA cm^−2^	[113]

Microwave synthesis can be performed as a standalone process or synergistically combined with other solution‐based methods, such as co‐precipitation^[^ ^106^, ^107^, ^109^ ^]^ and polymer‐precursor routes,^[^ ^108^ ^]^ enhancing precursor homogeneity and nucleation control. Typically, water‐soluble metal salts, commonly nitrates, sulfates, or chlorides, are dissolved in stoichiometric ratios and subjected to microwave irradiation. Non‐aqueous polar solvents may also be employed, provided they effectively absorb microwave energy through dipolar polarization, enabling efficient heating of the reaction medium. The resultant precipitates are usually filtered, washed, and dried, with optional post‐synthesis calcination to achieve desired crystallinity and phase purity.

**Figure 5:**
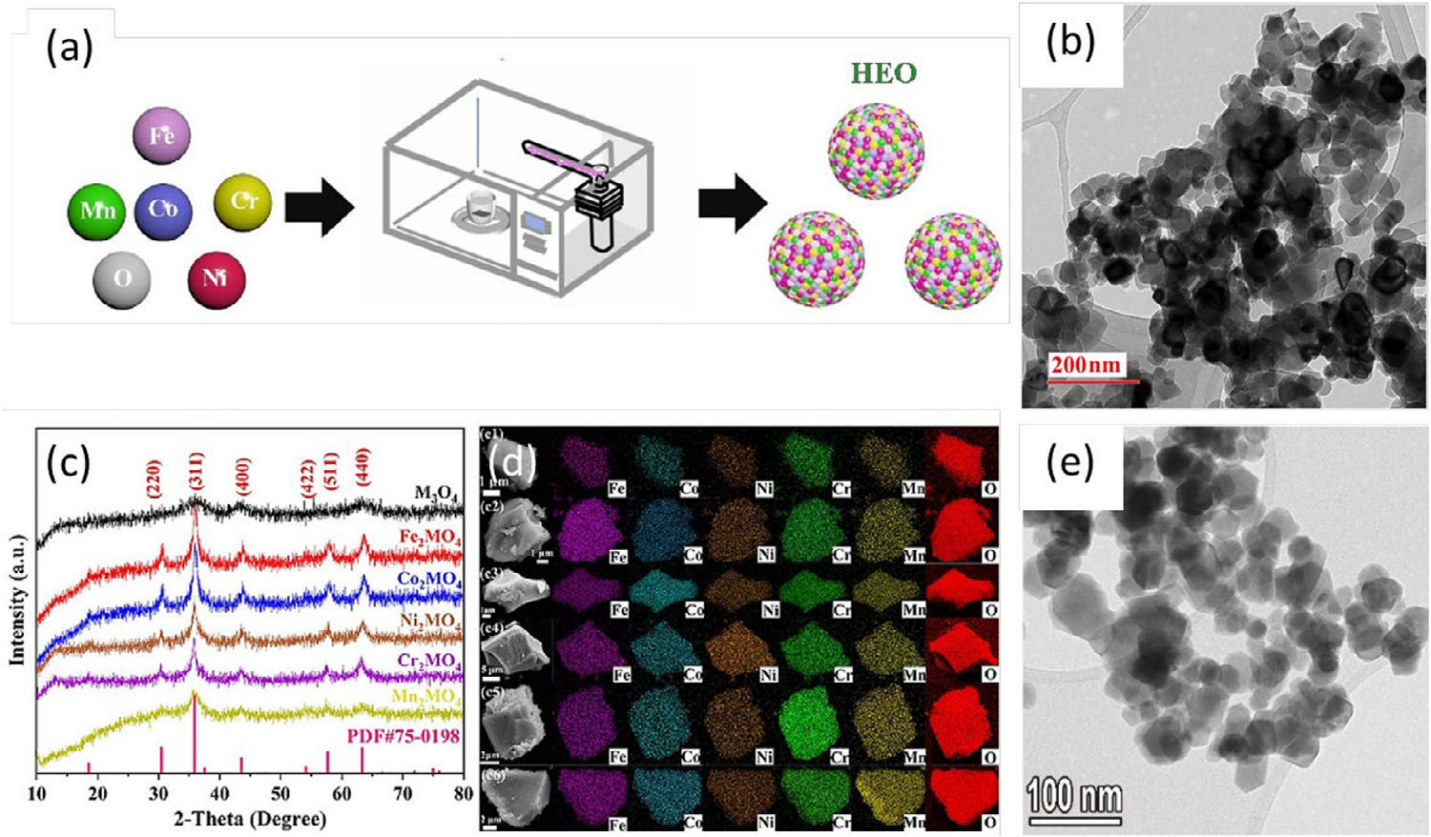
a) Microwave‐based synthesis schematic for HEOs. Adapted with permission.^[^ ^114^ ^]^ Copyright 2023, Elsevier. b) TEM micrograph of (Mg, Cu, Ni, Co, Zn)O powder calcined at 950 °C. Reproduced with permission.^[^ ^103^ ^]^ Copyright 2021, Elsevier. c) XRD patterns and (d) SEM‐EDS mapping of M_3_O_4_, (c2) Fe_2_MO_4_, (c3) Co_2_MO_4_, (c4) Ni_2_MO_4_, (c5) Cr_2_MO_4_, and (c6) Mn_2_MO_4_, with M = CoCrFeMnNi. Reproduced with permission.^[^ ^115^ ^]^ Copyright 2023, Elsevier. e) TEM micrograph of (Cr_0.2_Mn_0.2_Fe_0.2_Ni_0.2_Zn_0.2_)_3_O_4_. Reproduced with permission.^[^ ^109^ ^]^ Copyright 2023, Elsevier.

Microwave irradiation promotes uniform nucleation and crystal growth even at lower synthesis temperatures, contributing to narrower particle size distributions and suppressing elemental segregation or phase separation.^[^ ^111^, ^112^ ^]^ Representative microstructural analysis (see **Figure** **5**) illustrates the homogenous elemental distribution and consistent nanoscale particle sizes typical of microwave‐synthesized products. Owing to accelerated crystallization kinetics and enhanced mass‐transfer rates under microwave fields, crystallite sizes commonly range from 5 to 200 nm immediately after synthesis, with thermal annealing capable of increasing particle sizes beyond 500 nm.^[^ ^103^, ^106^, ^107^, ^108^, ^109^, ^113^ ^]^

Interestingly, microwave‐derived nanoparticles often exhibit optical band gaps lower than those obtained via conventional high‐temperature methods, an effect attributed to a higher concentration of lattice defects such as oxygen vacancies introduced during rapid synthesis.^[^ ^108^ ^]^ These defects play a dual role by facilitating enhanced incorporation of guest cations into the host lattice and promoting ionic transport, both critical factors for applications in catalysis and energy storage.^[^ ^116^, ^117^, ^118^ ^]^ Lin et al. have shown that such bulk defects can perturb molecular orbitals near the conduction band edge, thereby modulating electronic properties relevant for photocatalysis.^[^ ^119^ ^]^ Accordingly, microwave‐synthesized HEOs, such as (TiZrNbHfTa)O, demonstrate catalytic activities for CO_2_ reduction on par with state‐of‐the‐art photocatalysts, largely due to their elevated defect density and associated electronic structure modifications.^[^ ^120^ ^]^

The electrochemical performance of microwave‐synthesized HEOs has also been demonstrated by Kheradmandfard et al., who reported Co_0.2_Cu_0.2_Mg_0.2_Ni_0.2_Zn_0.2_O nanoparticles exhibiting reversible specific capacities exceeding 250 mAh g^−1^ at high charge/discharge rates of 5 A g^−1^ and excellent capacity retention (∼98%) over 1000 cycles at 1 A g^−1^, underscoring the method's potential for battery electrode material synthesis.^[^ ^103^, ^121^ ^]^ This performance notably surpasses that of analogous materials prepared by nebulized spray pyrolysis (NSP) as reported by Wang et al.^[^ ^103^, ^121^ ^]^ Furthermore, when employed as functional layers on sulfur cathodes in Li‐S cells, these microwave‐synthesized materials maintain high Coulombic efficiencies ≈99% over extended cycling (∼500 cycles), indicating their stability and electrochemical compatibility.^[^ ^106^ ^]^ High‐entropy sulfides can also be created with ultrafast synthesis times of 1 min at low temperatures below 300 °C using microwave synthesis. Highly crystalline spinel sulfides have been prepared as bifunctional electrocatalysts (HER and OER) for water splitting.^[^ ^122^ ^]^

In summary, microwave‐assisted synthesis offers a compelling route for the rapid and controlled production of HEMs with tunable nanostructures, defect profiles, and functional properties. Its ability to couple speed, energy efficiency, and compositional homogeneity makes it particularly attractive for scalable manufacturing of advanced materials for catalysis, energy storage, and electronic applications.

### 3.3 Sol–Gel Synthesis

Sol‐gel methods constitute a widely utilized approach for synthesizing high‐entropy ceramic nanoparticles, encompassing oxides, nitrides, and carbides. These processes enable precise control over composition, microstructure, and purity while typically operating at significantly lower temperatures than conventional solid‐state reactions.^[^ ^125^ ^]^ Nevertheless, sol‐gel synthesis is inherently sensitive to moisture, which can induce alterations in the resulting materials phase and structural characteristics, thereby necessitating stringent reaction conditions that may restrict its applicability, particularly for complex oxide systems. Moreover, the cost of sol‐gel precursors often exceeds that of traditional binary oxides, posing challenges for scalability in industrial contexts.^[^ ^126^, ^127^ ^]^

The sol‐gel synthesis typically proceeds through five sequential stages: hydrolysis, polycondensation, aging, drying, and calcination.^[^ ^128^ ^]^ Initially, metal alkoxide or metal salt precursors undergo hydrolysis in the presence of water, alcohol, or organic solvents, with oxygen playing a critical role in facilitating metal oxide nanoparticle formation (Equation 1). Aqueous sol‐gel routes utilize water as the reaction medium, whereas nonaqueous variants employ organic solvents with differing polarity profiles. Hydrolysis is commonly catalyzed by acidic or basic conditions, leading to the cleavage of metal–alkyl bonds according to the general reaction:

(1) $$M-\mathrm{OR}+H_{2}O\;\to \mathrm{MOH}+\mathrm{ROH}$$

where M denotes the metal center and R represents an alkyl group. Following hydrolysis, polycondensation reactions proceed by the elimination of water or alcohol molecules, linking metal hydroxide species to form extended networks (Equation 2):

(2) MOH+XO−M→MOM+XOH

where X can be hydrogen or an alkyl substituent. The presence of chelating agents, such as polyalcohols, facilitates the formation of cross‐linked polymeric networks, increasing solution viscosity and leading to gelation. The extent of water content directly influences the degree of network bridging. During the aging stage, further localized polycondensation reduces gel porosity and stabilizes the structure.^[^ ^125^ ^]^

**Figure 6:**
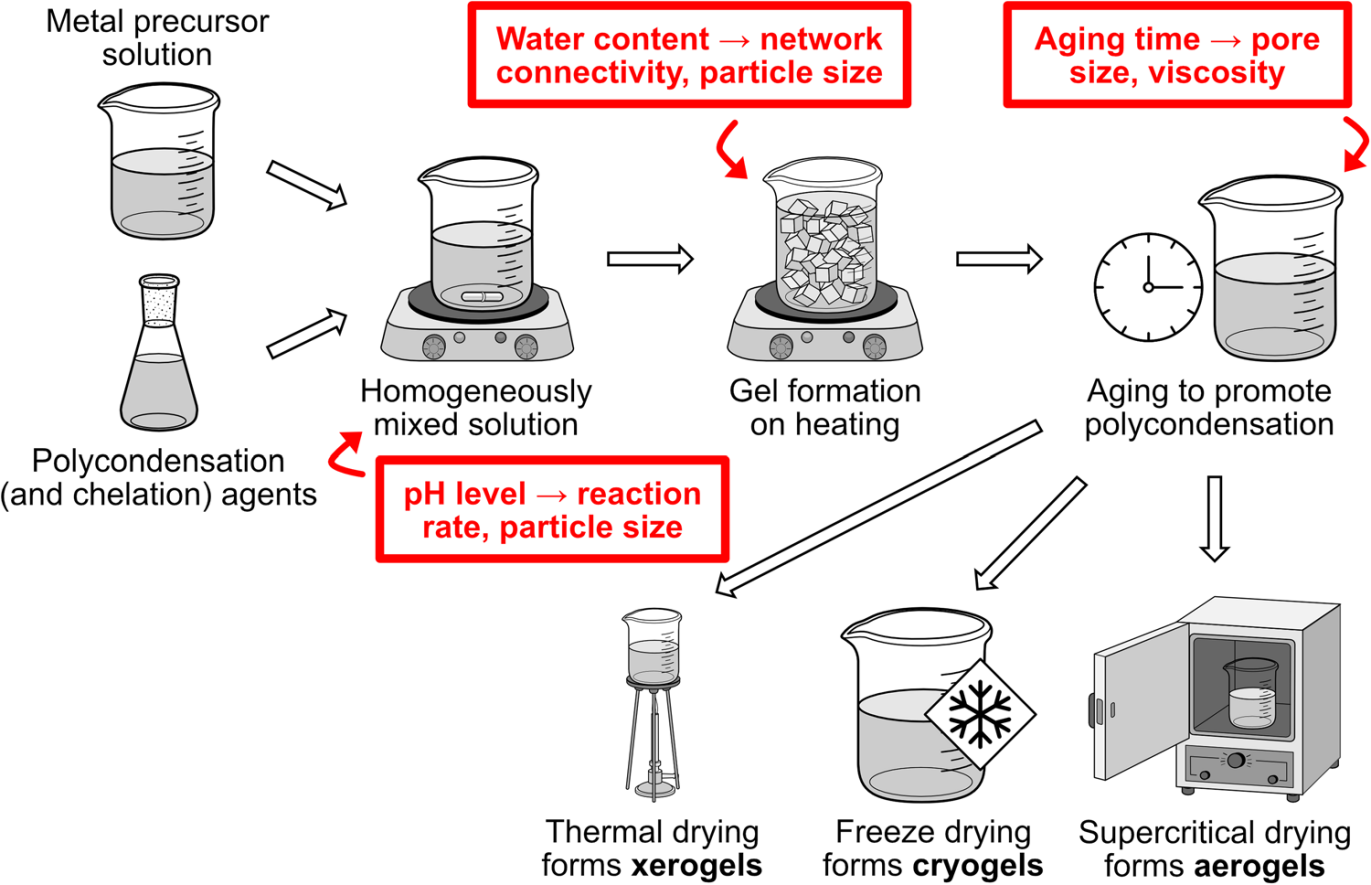
Schematic illustration of the sol‐gel synthesis route, highlighting key factors affecting material properties.

Drying is a critical phase, where different methods yield xerogels, aerogels, or cryogels, each possessing distinct porosity and density characteristics. Conventional thermal drying typically results in low‐porosity xerogels due to capillary stress during solvent evaporation. In contrast, supercritical drying preserves the gel's 3D network, producing highly porous aerogels, while freeze‐drying yields cryogels with intermediate microstructural features (**Figure** **6**).^[^ ^129^ ^]^ The final calcination step removes residual organics and volatile species, with temperature and duration profoundly impacting material density, grain growth, and pore evolution. Notably, identical crystalline phases can be achieved through either shorter, higher‐temperature calcinations or prolonged treatments at lower temperatures.^[^ ^130^ ^]^

Sol‐gel‐derived products frequently exhibit interconnected particle networks and porous channels, often resulting in lower specific surface areas relative to nanoparticles produced via other synthesis routes. This morphology can reduce efficacy in surface‐sensitive applications unless subjected to mechanical grinding prior to annealing. However, the inherent porosity and connectivity enable enhanced ion accommodation and oxygen vacancy formation, which bolster properties such as specific capacitance, redox kinetics, and electronic conductivity.^[^ ^94^, ^131^, ^132^, ^133^, ^134^ ^]^ Such oxygen vacancies and porous architectures render sol‐gel‐derived materials particularly advantageous for electrochemical energy storage, facilitating lithium‐ion and other charge carrier transport.^[^ ^135^, ^136^ ^]^ Furthermore, the sol‐gel method can substantially lower sintering temperatures; for example, the preparation of (FeCoNiCrMn)_3_O_4_ spinels requires a calcination temperature of 950 °C via sol‐gel synthesis compared to 1050 °C by conventional solid‐state routes.^[^ ^137^, ^138^ ^]^ The improved compositional uniformity and reduced thermal budget afforded by sol‐gel synthesis represent key advantages for manufacturing high‐quality high‐entropy ceramics.^[^ ^139^ ^]^ Reaction temperatures also play a role in determining the oxidation states of the constituent elements, which further affects the properties of the materials, for, e.g., in the aforementioned spinel, Mn is the only active site when acting as a catalyst during CO oxidation, thus making its oxidation state vital to the performance.^[^ ^140^ ^]^

A diverse spectrum of sol‐gel‐synthesized high‐entropy materials has been explored across multiple applications, including solid oxide fuel cells (SOFCs),^[^ ^141^ ^]^ supercapacitors,^[^ ^93^ ^]^ electrocatalysis,^[^ ^142^ ^]^ photocatalysis,^[^ ^143^ ^]^ rechargeable batteries,^[^ ^144^, ^145^ ^]^ general energy storage,^[^ ^146^ ^]^ and thermal barrier coatings.^[^ ^147^ ^]^ For instance, Jiang et al. demonstrated that calcination temperature critically influences grain size, porosity, elemental homogeneity, and surface defect density within the (Ga_0.2_Cr_0.2_Mn_0.2_Ni_0.2_Zn_0.2_)_3_O_4_ system. Higher annealing temperatures improved elemental distribution and increased surface defect concentrations while concomitantly reducing BET surface area due to pore closure, with optimized conditions yielding photocatalytic CO production rates surpassing 23 µmol h^−1^ g^−1^, exceeding many established catalysts.^[^ ^143^ ^]^

Organic polymers are frequently incorporated within sol‐gel matrices to direct particle morphology and porosity. Wang et al. successfully synthesized spherical mesoporous (NiCoCrFeMn)O particles exhibiting high specific surface areas ranging from 42 to 143 m^2^ g^−1^, enhancing catalytic and sensing functionalities.^[^ ^148^ ^]^ Similarly, Yang et al. reported that porous (Cr_0.2_Fe_0.2_Co_0.2_Ni_0.2_Zn_0.2_)_3_O_4_ prepared via sol‐gel exhibited remarkable electrochemical performance, delivering a high specific capacity of 1022 mAh g^−1^ after 1000 cycles at 1 A g^−1^, alongside excellent rate capability (220 mAh g^−1^ at 30 A g^−1^), attributed to improved ionic transport and electrolyte accessibility.^[^ ^145^ ^]^ **Table** **4** provides an overview of diverse high‐entropy materials fabricated by sol‐gel methods.

**Table 4:** Overview of HEOs and properties prepared by sol‐gel synthesis.

Formula	Structure	Application	Properties	Refs.
Mg_0.2_Co_0.2_Ni_0.2_Cu_0.2_Zn_0.2_O	Rock‐salt	Catalysis: CO oxidation	Resistance to deactivation by moisture in CO oxidation	[160]
Zr_0.5_Ti_0.5_Ce_0.5_Hf_0.5_O_7_	Pyrochlore	Energy storage: Supercapacitor	Specific capacitance of 703 F g^−1^ at 1 A g^−1^; Capacitance retention of ≈98% after 10 000 cycles; Increasing sintering temperature reduces the electrochemical performance	[132]
(La_0.2_Sr_0.2_Pr_0.2_Y_0.2_Ba_0.2_)(Co_0.2_Fe_0.8_)O_3−_ *_δ_*	Perovskite	Energy conversion: SOFC	Electrical conductivity of 100 S cm^−1^ at 550−700 °C; Oxygen reduction performance: 0.64 Ω cm^2^; A‐site elements majorly influence the performance	[135]
Ba(Sn_0.16_Zr_0.24_Ce_0.35_Y_0.1_Yb_0.1_Dy_0.05_)O_3−_ *_δ_*	Perovskite	Energy conversion: SOFC	Protonic conductivity of 8.3 mS cm^−1^ at 600 °C; Power density of 318 mW cm^−2^ at 600 °C	[149]
La(Mn_0.2_Fe_0.2_Co_0.2_Ni_0.2_Cu_0.2_)O_3−_ *_δ_*	Perovskite	Energy conversion: SOFC	Power density of 551 mW cm^−2^ at 800 °C	[161]
(La_0.7_Sr_0.3_)(Co_0.2_Cr_0.2_Fe_0.2_Mn_0.2_Ni_0.2_)O_3−_ *_δ_*	Perovskite	Energy conversion: SOFC	Power density of 550 mW cm^−2^ at 900 °C; Cathodic polarization resistance of ≈0.13 Ω cm^−2^ at 900 °C	[162]
(La_0.2_Nd_0.2_Sm_0.2_Ca_0.2_Sr_0.2_)MnO_3_	Perovskite	Energy conversion: SOFC	Electrical conductivity of ≈216 S cm^−1^ at 970 °C	[163]
(Cr_0.2_Mn_0.2_Fe_0.2_Co_0.2_Ni_0.2_)_3_O_4_	Spinel	Treatment: Capacitive deionization	Specific capacitances of 9−32 F g^−1^ at 5−100 mV s^−1^	[72]
(Ca*_x_* ZrYCeCr)O_2_	Fluorite	Catalysis: Piezo photocatalysis	Hydrogen production of 677 µmol g^−1^ h^−1^; Photocatalytic activity increased significantly by oxygen vacancies and defect sites	[164]
(Co_0.2_Ni_0.2_Mg_0.2_Zn_0.2_Mn_0.2_)Al_2_O_4_	Spinel	Catalysis: Methanation	Good stability for 320 h at temperatures up to 550 °C for CO_2_ methanation; Formation of a core‐shell structure using a reducing atmosphere	[165]
Ba_0.9_(Fe_0.2_Co_0.2_Ni_0.2_Zr_0.2_Y_0.2_)O_3−_ *_δ_*	Perovskite	Catalysis: Nitrogen reduction	Enhanced performance for electrocatalytic nitrogen reduction reaction due to increased oxygen vacancies	[166]
Li_1.8_(Cr_0.2_Mn_0.2_Fe_0.2_Co_0.2_Zn_0.2_)_3_O_4−_ *_δ_*	Spinel	Energy storage: Li‐ion anode	Specific capacity of 484 mAh g^−1^ after 300 cycles at 0.5 A g^−1^; Increase in oxygen vacancies to improve catalytic performance	[167]
(Cr_0.2_Mn_0.2_Fe_0.2_Co_0.2_Zn_0.2_)_3_O_4_/C	Spinel	Energy storage: Li‐ion anode	Specific capacity of 545 mAh g^−1^ after 400 cycles at 0.5 A g^−1^	[167]
(Cu_0.2_Ni_0.2_Fe_0.2_Co_0.2_Mg_0.2_)O*_x_*/Al_2_O_3_	Composite	Catalysis: CO oxidation	Superior SO_2_ tolerance; High stability in CO oxidation	[168]
La_0.6_Sr_0.4_Co_0.2_Fe_0.2_Mn_0.2_Ni_0.2_Mg_0.2_O_3_	Perovskite	Catalysis: OER	Overpotential of 320 mV at 10 mA cm^−2^	[169]
(FeCoMnZnMg)_3_O_4_	Spinel	Catalysis: OER	Overpotential of 240 mV at 10 mA cm^−2^; Tafel slope of 59 mV dec^−1^; Durability of 1000 h without any potential loss; Mesoporous structure formed due to gas evolution (increasing specific surface area)	[170]

High‐entropy perovskites synthesized through sol‐gel techniques have shown promise for SOFC applications, with some compositions, such as (La_0.6_Sr_0.4_)(Co_0.2_Fe_0.8_)O_3‐_ *_δ_*, outperforming conventional counterparts.^[^ ^135^, ^149^ ^]^ These sol‐gel‐derived rare‐earth perovskites exhibit enhanced proton conductivity, driven by oxygen vacancy formation, while pyrochlore‐structured rare‐earth oxides afford superior corrosion resistance and thermal insulation at temperatures exceeding 1200 °C.^[^ ^150^, ^151^, ^152^ ^]^

To improve precursor stoichiometry and stability, chelating agents like citric acid or ethylenediaminetetraacetic acid (EDTA) are routinely employed, facilitating the synthesis of metals otherwise challenging to incorporate due to unstable hydroxo species, such as Ti and Nb.^[^ ^153^, ^154^, ^155^ ^]^ An example of this strategy is the Pechini method, which employs polymer networks to complex metal cations and was recently adapted by Dąbrowa et al. in 2021 to synthesize high‐entropy perovskite oxides such as Ln_1‐_ *_x_* Sr*_x_* (CoCrFeMnNi)O_3‐_ *_δ_*.^[^ ^141^ ^]^ Although less common, this approach has demonstrated notable potential for producing perovskite and spinel materials targeted for energy applications, microdevices, and thermal barrier coatings.^[^ ^156^, ^157^, ^158^, ^159^ ^]^

### 3.4 Solution‐Combustion Synthesis

**Figure 7:**
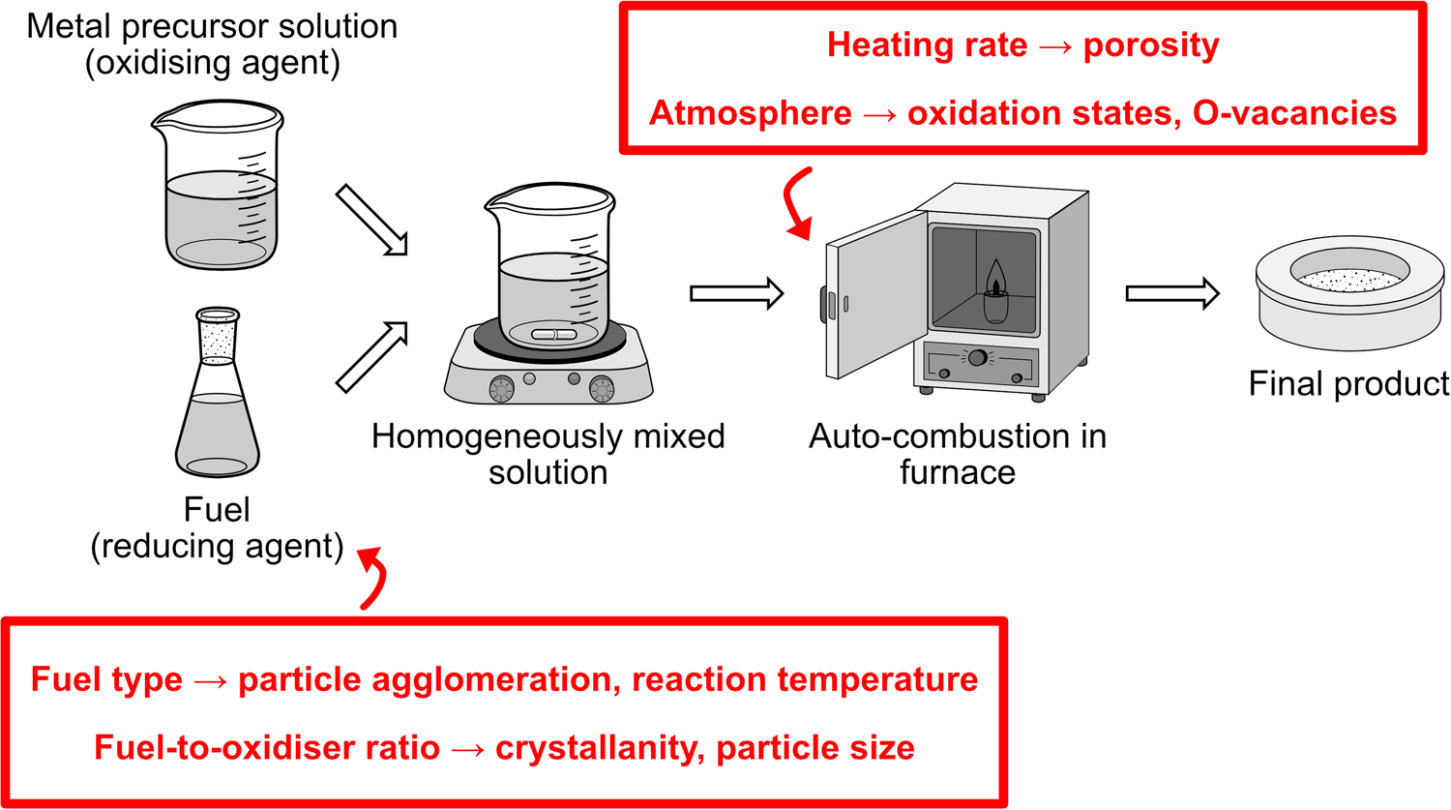
Schematic illustration of the solution combustion synthesis route, highlighting key factors affecting material properties.

Solution‐combustion synthesis (SCS) represents a combustion‐driven technique characterized by a rapid, self‐sustaining exothermic reaction that facilitates the formation of highly crystalline materials within short timeframes. Rooted in self‐propagating high‐temperature synthesis principles,^[^ ^171^ ^]^ SCS achieves ignition via a swift exothermic reaction among precursor components, enabling direct formation of the target phase. Kingsley and Patil notably adapted this method for metal oxide production by employing metal salts, commonly nitrates, sulfates, or carbonates, as oxidizing agents, while organic fuels such as urea, glycine, starch, citric acid, or carbohydrazide serve as reducing counterparts (**Figure** **7**).^[^ ^172^ ^]^

This approach confers several distinct advantages, including high product purity, energetic efficiency, and tunable reaction conditions. The stoichiometry and chemical nature of the fuel and oxidizer critically influence combustion and product characteristics. Metal nitrates are preferred precursors owing to their excellent solubility, relatively low decomposition temperatures, and inherent oxidizing capability.^[^ ^173^ ^]^ Among fuels, glycine and urea are favored due to their affordability, availability, significant exothermicity, and capacity to form metal‐nitrate complexes, which aid in homogeneous mixing and combustion control.^[^ ^174^ ^]^ The fuel‐to‐oxidizer ratio (*ϕ*), defined as the ratio of total valency of fuel to oxidizer (Equation 3), serves as a pivotal parameter regulating combustion intensity, gas evolution, and consequently, microstructural evolution:^[^ ^175^ ^]^

(3) $$\phi =\frac{\mathrm{total}\;\mathrm{valency}\;\mathrm{of}\;\mathrm{fuel}}{\mathrm{total}\;\mathrm{valency}\;\mathrm{of}\;\mathrm{oxidizer}}$$

Elevated *ϕ* promote greater gas release during combustion, which enhances porosity and surface area through the creation of voluminous gas bubbles, but simultaneously may reduce crystallinity due to rapid quenching effects. Conversely, lower *ϕ* ratios favor increased crystallinity but often at the expense of decreased porosity. Excess fuel facilitates the formation of smaller particles with higher BET surface areas, yet overly reductive environments induced by surplus fuel can lead to the emergence of secondary or undesirable phases.^[^ ^176^, ^177^ ^]^ The final SCS products are typically composed of interconnected nanoparticles aggregated into micrometre‐scale clusters.^[^ ^177^, ^178^ ^]^ Temperature control during combustion is critical; for example, temperatures below 850 °C result in incomplete phase integration of ZnO (wurtzite) and CuO (tenorite) within rock‐salt structured high‐entropy oxides.^[^ ^179^ ^]^ Another parameter that can cause changes within the phase stability of HEOs is the reaction pressure, particularly the partial pressure of oxygen. High pressure can cause metastable phases like modified ludwigite‐type —(Cr,Mn,Fe,Co,Ni)_4_O_5_— to form, which cannot be obtained under normal reaction conditions.^[^ ^180^ ^]^

SCS inherently produces highly porous materials enriched with structural defects due to vigorous gas evolution during the exothermic reaction. The resulting pore network not only inhibits particle agglomeration but also generates a large specific surface area, conducive to catalytic site dispersion, efficient reaction kinetics, and enhanced interfacial transport phenomena.^[^ ^181^ ^]^ High‐entropy oxides synthesized by SCS can be engineered to contain elevated concentrations of oxygen vacancies, which significantly boost catalytic activity and electrochemical performance. For instance, the (Fe_0.2_Zn_0.2_Co_0.2_Ni_0.2_Cu_0.2_)Fe_2_O_4_ spinel exhibits enhanced selectivity toward H_2_O_2_ generation, attributed to such defect structures.^[^ ^182^ ^]^ Similarly, the presence of abundant oxygen vacancies in (Co_0.2_Cr_0.2_Fe_0.2_Mn_0.2_Ni_0.2_)_3_O_4_ increases Li‐ion accommodation sites, thereby improving electronic conductivity and ion migration critical for battery applications.^[^ ^136^ ^]^ These properties underscore the suitability of SCS‐derived materials for diverse applications, including catalysis, energy storage, sensing, and adsorption technologies.^[^ ^183^ ^]^ **Table** **5** summarizes recent examples of high‐entropy oxides prepared via solution‐combustion methods.

**Table 5:** Overview of HEOs and properties prepared by solution‐combustion synthesis.

Formula	Structure	Application	Properties	Refs.
(Fe_0.2_Zn_0.2_Co_0.2_Ni_0.2_Cu_0.2_)Fe_2_O_4_	Spinel	Catalysis: Oxygen reduction reaction (ORR)	Selectivity of 85% for H_2_O_2_ production due to high concentration of oxygen vacancies	[182]
(La_0.2_Pr_0.2_Nd_0.2_Sm_0.2_Ba_0.1_Sr_0.1_)(Co_0.2_Fe_0.6_Ni_0.1_Cu_0.1_)O_3−_ *_δ_*	Perovskite	Energy conversion: SOFC	Electrical conductivity of 635.2 S cm^−1^ at 800 °C; Power density of 715 mW cm^−2^ at 800 °C	[185]
(La_0.2_Pr_0.2_Nd_0.2_Sm_0.2_Sr_0.2_)MnO_3−_ *_δ_*	Perovskite	Energy conversion: SOFC	Suppressing Sr segregation in the cathode	[186]
(Co_0.2_Cr_0.2_Fe_0.2_Mn_0.2_Ni_0.2_)_3_O_4_	Spinel	Energy storage: Li‐ion anode	Specific capacity of 428 mAh g^−1^ at 10 A g^−1^; Energy density of 372 Wh kg^−1^ (full cell with LiFePO_4_ cathode); High concentration of oxygen vacancies lead to high rate performance	[136]
(FeNiCrMnMgAl)_3_O_4_	Spinel	Energy storage: Li‐ion anode	Specific capacity of 670 mAh g^−1^ after 200 cycles at 0.2 A g^−1^	[187]
(FeCoNiMoRu)_3_O_4_	Spinel	Catalysis: OER	Overpotential of 199 mV at 10 mA cm^−2^, and 266 mV at 100 mA cm^−2^; Tafel slope of 40 mV dec^−1^; Excellent long‐term stability (operating at 500 mA cm^−2^ for 100 h); Large active surface area can be achieved due to formation of nanosheets	[188]

The SCS technique has also been successfully applied to synthesize well‐established high‐entropy oxides such as Ce_0.2_Gd_0.2_Sm_0.2_Y_0.2_Zr_0.2_O_2−_ *_δ_* at lower temperatures and in significantly reduced synthesis durations compared to conventional routes, without observable detriment to material properties.^[^ ^184^ ^]^ This underscores the ability of SCS to efficiently produce high‐quality materials with distinct chemical and physical characteristics under relatively mild external thermal conditions.

Theoretical investigations employing thermodynamic modelling have been instrumental in optimizing fuel selection and stoichiometry, facilitating precise control over combustion kinetics and phase formation. Adjusting activity coefficients and fuel consumption rates enables the synthesis of single‐phase high‐entropy oxides with tailored physicochemical properties.^[^ ^177^ ^]^ Leveraging these insights, researchers have engineered high‐entropy oxides exhibiting superior crystallinity, specific surface area, and thermal stability by judiciously selecting fuel types and fuel‐lean combustion regimes. Notably, powders synthesized using glycine under fuel‐lean conditions demonstrate enhanced performance metrics, providing critical guidance for the targeted design and modification of HEMs for catalytic applications.

### 3.5 Hydrothermal and Solvothermal Synthesis

Hydrothermal and solvothermal synthesis techniques represent versatile, low‐energy, and highly controllable approaches for the preparation of nanostructured materials, including high‐entropy oxides and frameworks. These methods involve the chemical transformation of solvated metal precursors within a sealed vessel, typically a Teflon‐lined autoclave, under elevated pressures and relatively moderate temperatures. Reaction temperatures typically range from 100 to 300 °C, with internal pressures arising autogenously from the vaporized solvents. Although the reaction times are often prolonged compared to other synthesis routes, these methods offer exceptional control over crystallinity, morphology, particle size, and compositional homogeneity by fine‐tuning parameters such as temperature, pressure, reaction time, precursor concentration, pH, and additive or surfactant composition.^[^ ^189^ ^]^

A key advantage of hydro‐/solvothermal synthesis lies in the liquid‐phase environment, which promotes enhanced ion diffusion rates and facilitates homogeneous mixing of multiple metal ions at the atomic level. This feature enables direct incorporation of diverse cations into a single‐phase high‐entropy lattice without the need for a high‐temperature calcination step.^[^ ^190^ ^]^ As such, it avoids the complex lattice deconstruction and reconstruction processes often required in traditional solid‐state reactions. The use of surfactants such as cetyltrimethylammonium bromide (CTAB) can further lower synthesis temperatures by stabilizing specific facets of nucleating crystallites. For instance, in the synthesis of (FeCoNiCrMn)_3_O_4_, the incorporation of CTAB allowed the reaction temperature to be reduced to 900 °C while maintaining phase purity and structural integrity.^[^ ^191^ ^]^ More complex structures like layered P2 with a high percentage of active facets are also easily synthesized with a hydrothermal route. These provide a stable pathway for ion migration, helping improve the electrochemical kinetics when used as electrodes materials for energy storage.^[^ ^192^ ^]^

This intrinsic adaptability makes hydrothermal and solvothermal methods especially suitable for the synthesis of HEMs incorporating thermally sensitive species, such as organic ligands or soft anions. Consequently, these techniques have emerged as powerful tools for producing high‐entropy metal‐organic frameworks (HE‐MOFs).^[^ ^193^ ^]^ These systems combine structural diversity with modularity in metal‐ligand selection, enabling the fine‐tuning of porosity, functionality, and elemental homogeneity. Xu et al. successfully demonstrated the synthesis of a HE‐MOF composed of five isoperiodic transition metals with high ligand coordination compatibility. The resulting framework displayed uniform elemental dispersion and phase purity, highlighting the potential of this approach for advanced structural control at the molecular level.^[^ ^194^ ^]^ High‐entropy sulfoselenides like Cu_0.88_Sn_0.02_Sb_0.02_Bi_0.02_Mn_0.02_S_0.9_Se_0.1_ have also been synthesized for use as electrode materials in Na‐ion and Li‐ion batteries. These materials show improved performance due to their chemical and structural stability, reducing volume expansion, cracking, and surface reactions.^[^ ^195^ ^]^

In addition to direct synthesis, hydro‐/solvothermal methods also serve as pre‐synthetic or intermediate steps within templated approaches. Combining these techniques with post‐treatment processes, such as calcination or pyrolysis, enables the transformation of precursor phases into tailored architectures with enhanced functionality. For instance, Miao et al. employed a HE‐MOF as a self‐sacrificial template to construct a hollow polyhedral oxide structure (ZnFeNiCuCoRu‐O), exhibiting excellent catalytic performance across the full pH range due to its large surface area and accessible active sites.^[^ ^196^ ^]^ This synergy between hydrothermal synthesis and template‐guided morphology control opens new avenues for designing materials with hierarchical porosity and structural anisotropy, essential for catalytic and energy‐related applications.

Recent advances have extended these synthesis strategies toward scalable production. The first successful attempts to upscale the hydrothermal/solvothermal synthesis of HEMs demonstrate the feasibility of industrial applications, paving the way for the integration of these materials into real‐world technologies.^[^ ^197^ ^]^ The relatively mild reaction conditions, high reproducibility, and compositional tunability make these methods particularly attractive for synthesizing high‐entropy ceramics, MOFs, and composite materials where both structural precision and compositional diversity are required.

### 3.6 Functional Implications of Solution‐Based Synthesis

**Figure 8:**
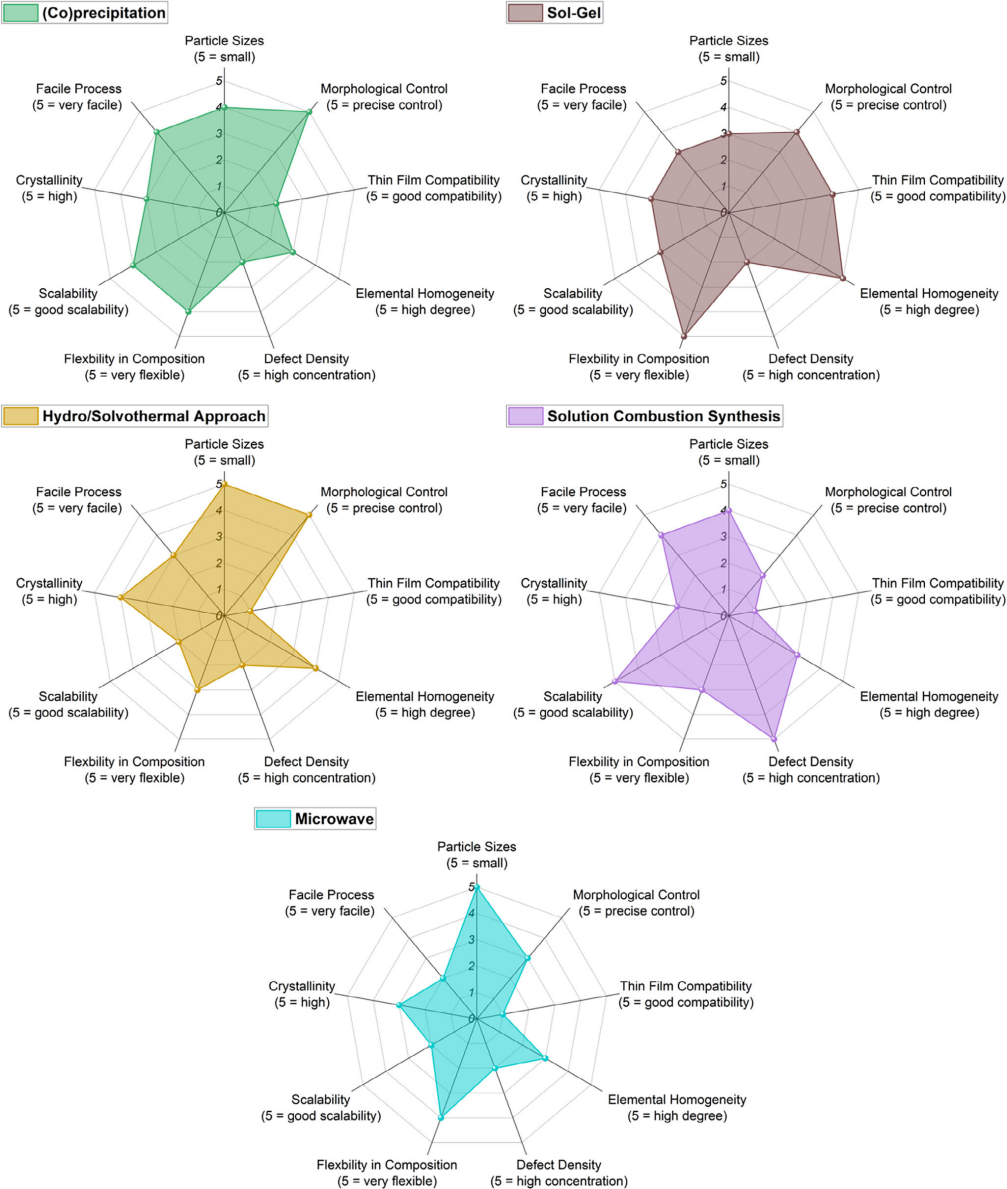
Radar charts for solution‐based synthesis methods.

Solution‐based synthesis techniques, including co‐precipitation, sol‐gel, microwave‐assisted, solution‐combustion, and hydro‐/solvothermal methods, offer a highly tunable and versatile toolbox for producing HEMs with controlled composition, size, shape, and crystallinity. Unlike solid‐state and mechanochemical routes, which are largely governed by diffusion‐limited kinetics or mechanical energy input, solution‐based processes are driven by precise control of chemical equilibria and nucleation–growth dynamics in the liquid phase. This fundamental difference enables the targeted engineering of structural and morphological features that are directly linked to key functional properties in diverse applications (**Figure** **8**), such as catalysis, batteries, sensors, and thin‐film devices.

#### 3.6.1 Particle Sizes

Solution‐based synthesis approaches typically offer excellent control over particle size in the nanometer to sub‐micron range. Hydrothermal and solvothermal methods, in particular, allow the tailoring of size by adjusting temperature, pressure, pH, and mineralizers. Sol‐gel and (co) precipitation methods also enable the formation of ultrasmall particles due to the molecular‐level mixing of precursors. In contrast, solution combustion and microwave synthesis often result in rapid nucleation and growth, sometimes producing slightly larger or polydisperse particles. Nonetheless, their product sizes are still significantly smaller than those achieved through solid‐state routes.

#### 3.6.2 Morphological Control

Among solution‐based routes, hydro‐/solvothermal and sol‐gel methods stand out for their ability to produce tailored morphologies such as rods, sheets, spheres, and even hollow or hierarchical structures. These morphologies can be directed through surfactants, templating agents, or the use of structure‐directing solvents. In (co)precipitation and microwave‐assisted syntheses, morphology is more difficult to control and often results in isotropic, less‐defined structures. Solution combustion synthesis tends to yield foam‐like or highly porous structures due to the rapid gas evolution, which can be advantageous for applications like catalysis.

#### 3.6.3 Thin‐Film Compatibility

Some solution‐based approaches, especially sol‐gel and (co)precipitation, can be adapted for thin‐film deposition via techniques like spin coating, dip coating, or spray deposition. These methods allow for uniform coatings on a variety of substrates. Hydrothermal synthesis has also been applied to grow films directly on conductive or porous. In contrast, solution combustion and microwave synthesis are less frequently used for thin films, though recent advances in combustion‐derived ink formulations have begun to bridge this gap.

#### 3.6.4 Elemental Homogeneity

High elemental homogeneity is one of the strongest advantages of solution‐based synthesis. Mixing takes place at the molecular or ionic level, allowing for uniform distribution of multiple cations even in high‐entropy systems. Sol‐gel and (co)precipitation methods are particularly suited for achieving excellent homogeneity. Hydrothermal and solvothermal syntheses also maintain good compositional uniformity, although segregation may occur if the solubility products or reaction rates of precursors vary significantly. Rapid synthesis routes like solution combustion or microwave methods require careful tailoring of precursor reactivity to avoid local compositional inhomogeneities.

#### 3.6.5 Defect Density

Low‐temperature processing in most solution‐based methods inherently introduces point defects, vacancies, or structural distortions, features that can be beneficial or detrimental depending on the application. For example, oxygen vacancies in transition metal oxides synthesized via sol‐gel or combustion routes can enhance catalytic performance. On the other hand, excessive defects from incomplete crystallization in (co)precipitated or microwave‐processed materials may impair stability or electronic conductivity. Post‐synthetic treatments (e.g., annealing) are often used to optimize the defect profile.

#### 3.6.6 Flexibility in Composition

Solution‐based synthesis techniques are exceptionally versatile in accommodating a wide variety of metal precursors, including those with different valences, ionic radii, and reactivity. This makes them particularly suitable for the synthesis of HEMs. Sol‐gel and (co)precipitation are highly, while hydrothermal methods can be limited by the solubility and hydrolysis behavior of certain ions. Microwave and combustion methods require careful balancing of precursor energetics, but still support multicomponent systems with proper design.

#### 3.6.7 Scalability

Many solution‐based methods are inherently scalable. (Co)precipitation and sol‐gel syntheses are already used industrially for the production of ceramic powders and catalysts. Hydrothermal methods are more difficult to scale due to the need for high‐pressure vessels and long reaction times, although continuous‐flow hydrothermal reactors offer a promising route. Microwave synthesis is limited by penetration depth and reactor size. Solution combustion synthesis can be scaled for batch processes, but controlling heat release and uniformity becomes challenging at larger volumes.

#### 3.6.8 Crystallinity

Crystallinity varies strongly depending on the synthesis route and conditions. Hydrothermal and solvothermal methods typically yield well‐crystallized products at relatively low temperatures, especially when long reaction times are used. Sol‐gel and (co)precipitation methods usually produce amorphous or poorly crystalline materials that require post‐synthetic calcination to achieve full crystallinity. Microwave synthesis can rapidly induce crystallization, though the resulting crystallinity is often moderate. Solution combustion synthesis tends to result in nanocrystalline phases due to the extreme temperatures reached during combustion, but crystal quality may vary.

#### 3.6.9 Process Simplicity

(Co)precipitation and sol‐gel synthesis are among the most facile and most accessible routes, relying on common precursors, mild conditions, and straightforward protocols. Hydrothermal and solvothermal methods require more specialized equipment and parameter optimization. Microwave‐assisted synthesis is relatively simple in setup, though it demands careful control over microwave absorption and reaction vessel compatibility. Solution combustion is fast and energetically efficient, but it poses safety concerns due to the exothermic nature of the reaction and gas evolution.

## 4 Gas‐Phase and Aerosol‐Based Synthesis Methods

Gas‐phase and aerosol‐based synthesis techniques offer a distinct approach to nanoparticle fabrication, relying on the conversion of vapor‐phase precursors into condensed solid particles through nucleation, growth, and subsequent agglomeration processes. These methods are particularly effective when the vapor phase is thermodynamically less stable than the corresponding solid phase, creating favorable conditions for the spontaneous formation of nuclei from a supersaturated vapor. Upon nucleation, particles grow through condensation and coagulation mechanisms, which are influenced by the chemical composition of the vapor, the degree of supersaturation, and external process parameters such as temperature and pressure.

Once formed, nanoparticles undergo random motion via Brownian diffusion, leading to frequent collisions. These collisions result in coagulation driven by van der Waals and electrostatic interactions.^[^ ^198^ ^]^ The evolution of particle size and morphology during this stage depends on the interplay between coagulation and coalescence. While coagulation increases the particle size through aggregation, coalescence, governed by the sintering behavior of contacting particles, leads to densification and shape transformation. At elevated temperatures, coalescence is accelerated, producing nearly spherical, compact particles, whereas at lower temperatures, limited sintering preserves loosely bound, irregular aggregates. Intermediate conditions may result in partially sintered, fractal‐like morphologies. Thus, temperature control is essential to tuning the final particle shape and density, especially when targeting either soft agglomerates or discrete nanoparticles.^[^ ^199^, ^200^, ^201^, ^202^ ^]^

Gas‐phase synthesis routes generally produce smaller primary particles compared to solid‐state or solution‐based methods.^[^ ^203^ ^]^ Furthermore, the absence of solvents and the inherently clean reaction environment minimize the risk of contamination, making this approach ideal for synthesizing high‐purity nanomaterials. The reactor configuration, gas flow rates, and temperature profiles significantly influence nucleation and growth rates, enabling precise control over particle size distribution, morphology, crystallinity, and composition.^[^ ^204^ ^]^ Reactor residence time and precursor concentration further modulate supersaturation levels, which determine nucleation density and growth kinetics.^[^ ^200^ ^]^ These parameters can be optimized to yield highly uniform, monodisperse particles with tailored characteristics.^[^ ^205^, ^206^, ^207^ ^]^

**Figure 9:**
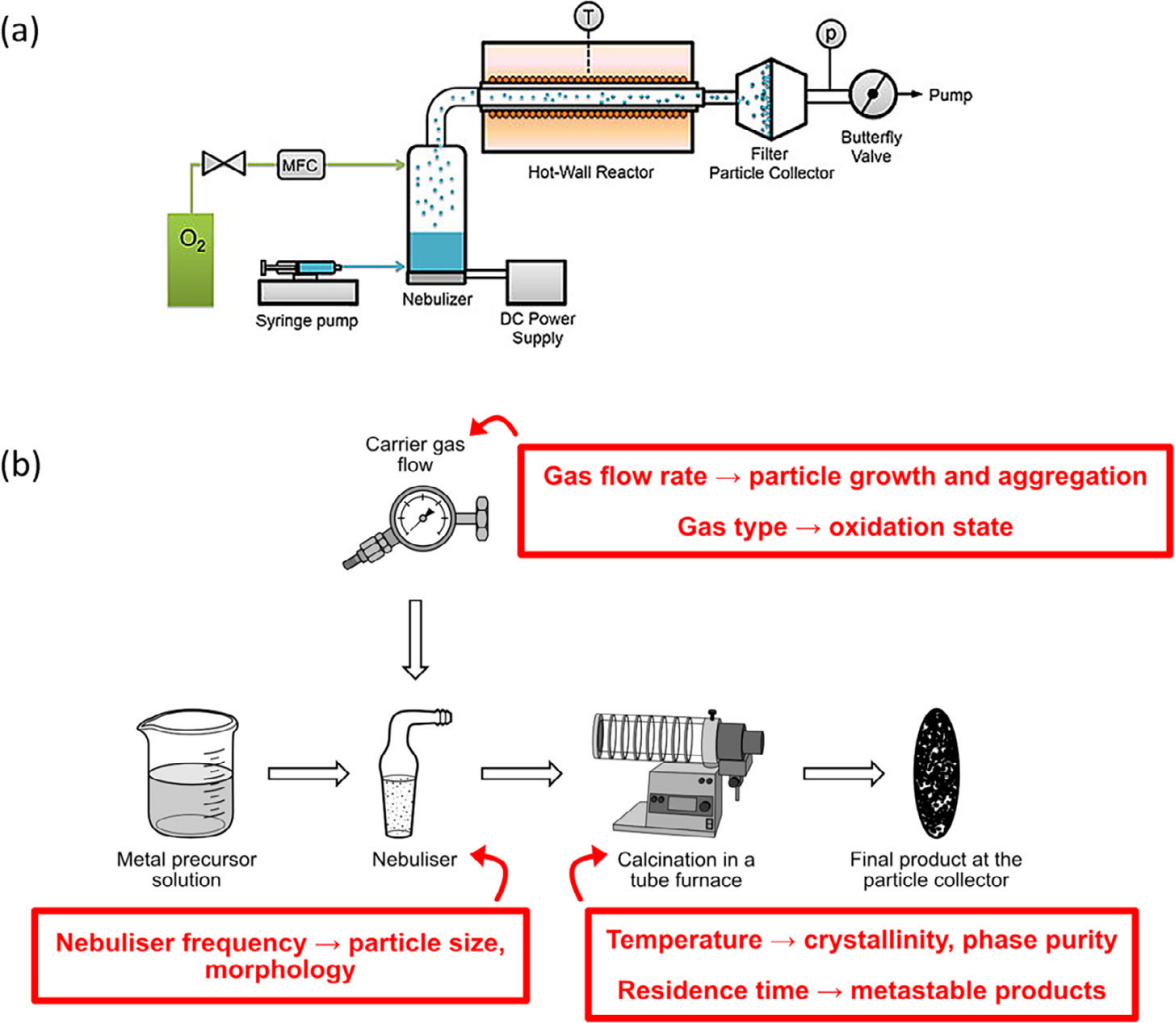
a) Schematic illustration of the nebulized spray pyrolysis synthesis route. Reproduced with permission.^[^ ^216^ ^]^ Copyright 2014, Elsevier. b) Highlighting the key factors affecting material properties.

Among aerosol‐based methods, flame spray pyrolysis (FSP) and nebulized spray pyrolysis (NSP) have gained attention for synthesizing multicomponent oxides and nanostructured ceramics (**Figure** **9**). NSP, in particular, has been applied for the synthesis of high‐entropy oxides by atomizing a precursor solution into fine droplets, which are transported through a high‐temperature zone where solvent evaporation, precursor decomposition, and particle formation occur in rapid succession. This technique offers high compositional tunability and continuous production, making it attractive for scaling up HEMs with homogeneous elemental distributions.

Despite the promise of gas‐phase synthesis for HEMs, its application remains limited due to challenges in maintaining compositional uniformity when multiple elements with different vapor pressures and chemical reactivities are involved. Advanced precursor design, such as the use of metal‐organic complexes with matched decomposition temperatures, can help to overcome these issues and enable co‐nucleation of multiple elements. For example, studies utilizing carefully formulated precursor mixtures have successfully synthesized high‐entropy rock‐salt and spinel‐type nanoparticles with controlled size and structure.

Overall, gas‐phase and aerosol‐based synthesis methods represent powerful, solvent‐free techniques that enable continuous, scalable fabrication of high‐purity, nanostructured HEMs. The unique ability to fine‐tune particle properties through careful control of thermodynamic and kinetic parameters makes these methods especially appealing for applications requiring tight control over size, crystallinity, and morphology, such as in catalysis, gas sensing, or thin‐film deposition.

### 4.1 Chemical Vapor Deposition and Nebulized Spray Pyrolysis

Chemical vapor synthesis (CVS), a subclass of chemical vapor deposition (CVD), is a gas‐phase route for the preparation of nanocrystalline ceramic powders. In this process, volatile metal‐organic precursors are transported in the vapor phase into a hot‐wall reactor, where they undergo thermal decomposition or react with an introduced reactive gas to form condensed solid products. The nature of the final product, whether in the form of thin films or particulate matter, is largely governed by the reactor's temperature profile, pressure, and gas flow parameters, particularly the residence time of the precursor vapor within the hot zone. A longer residence time typically promotes deposition onto the reactor walls, favoring thin‐film formation, whereas faster gas flow rates and optimized precursor volatility can shift the process toward homogeneous nucleation and nanoparticle formation. Chang et al. demonstrated that by adjusting reactor pressure, precursor concentration, and flow dynamics, the process can be directed away from wall deposition and toward the synthesis of discrete nanopowders with controlled composition and morphology.^[^ ^208^ ^]^

CVS has evolved into a robust platform for producing a wide range of nanostructured ceramic materials, including nitrides, carbides, oxides, and sulfides. Compared to inert gas condensation (IGC), CVS offers improved control over particle stoichiometry, crystallinity, and uniformity. Key parameters influencing CVS include the choice of precursors (particularly their decomposition temperature and volatility), the length and thermal gradient of the hot zone, and the partial pressures of each reactive component.^[^ ^205^, ^209^, ^210^ ^]^ These variables can be tuned to synthesize single‐phase, doped, or multiphase nanoparticles. More recently, aerosol‐assisted CVD has been developed to deposit high‐entropy sulfide thin films with uniform composition and tailored electronic properties.^[^ ^211^ ^]^

Nebulized spray pyrolysis (NSP) represents a modular and scalable variant of CVS. In this method, a liquid precursor solution, typically containing a homogeneous mixture of metal nitrates or organometallic compounds, is converted into an aerosol using an ultrasonic nebulizer or pneumatic atomizer. The resulting droplets are carried by an inert or reactive gas stream (such as N_2_, Ar, or O_2_) into a heated reactor where rapid solvent evaporation and intradroplet thermal decomposition lead to the formation of solid oxide nanoparticles. The process is particularly amenable to multicomponent systems, as the co‐localization of metal cations within each droplet ensures uniform distribution in the resulting particle. This has enabled the successful synthesis of high‐entropy oxides (HEOs) incorporating up to ten different elements into a single rock‐salt or perovskite‐type phase.^[^ ^14^, ^36^, ^58^, ^212^, ^213^, ^214^ ^]^

NSP provides numerous advantages, including continuous processing, ease of compositional tuning, and compatibility with aqueous precursors. Moreover, the combination of high precursor throughput, short reaction times, and rapid quenching allows for fine control over particle size and phase evolution. Despite these benefits, achieving uniform nanoscale particles (<100 nm) with high crystallinity across the entire product batch remains a challenge, particularly in complex systems with significant ionic radii differences or mixed valence states.

Flame spray pyrolysis (FSP) offers an alternative aerosol‐based method wherein the precursor aerosol is injected directly into a flame. The intense flame temperatures (>1900 °C) induce rapid combustion of the precursor and immediate nucleation of the product particles. The short residence times and steep thermal gradients prevent excessive sintering, preserving the nanostructured nature of the material. Unlike NSP, FSP allows the use of non‐volatile or thermally unstable precursors dissolved in flammable solvents. This enables the incorporation of low‐vapor‐pressure or otherwise incompatible elements into the final product. For example, Phakatkar et al. synthesized oxygen‐deficient (MnFeNiCuZn)_3_O_4_ nanoparticles via FSP and observed preferential site occupation of higher‐valent cations in the spinel lattice.^[^ ^215^ ^]^ Brandt et al. further demonstrated that FSP‐derived HEOs, such as (ZnNiMnFeTi)_3_O_4_, exhibit improved performance in lithium‐ion batteries while avoiding the use of critical raw materials like cobalt.^[^ ^24^ ^]^

Both NSP and FSP benefit from the rapid heating and cooling intrinsic to aerosol‐based processing, which can stabilize metastable phases and fine‐tune defect structures. These dynamic thermal environments promote homogeneous nucleation and suppress undesired phase segregation, offering pathways to engineer high‐entropy compositions with optimized electrochemical or catalytic performance. However, maintaining control over agglomeration, elemental homogeneity, and particle size distribution at larger production scales remains challenging.^[^ ^58^ ^]^

In summary, CVS, NSP, and FSP constitute versatile and scalable synthesis routes for high‐entropy ceramics and nanomaterials (representative materials and their key properties are provided in **Table** **6**). Their adaptability to various precursor chemistries and capacity for continuous production make them strong candidates for industrial translation. When carefully optimized, these techniques offer unique capabilities for controlling particle morphology, crystallinity, and compositional uniformity—properties that are closely tied to the functional performance of HEMs in electronic, catalytic, and energy storage applications.

**Table 6:** Overview of HEOs and properties prepared by spray pyrolysis synthesis.

Formula	Structure	Application	Properties	Refs.
Co_0.2_Cu_0.2_Mg_0.2_Ni_0.2_Zn_0.2_O	Rock‐salt	Energy storage: Li‐ion anode	Specific capacity of ≈600 mAh g^−1^ after 100 cycles at 0.2 A g^−1^; Energy density of 236 Wh kg^−1^	[14, 121]
(Ce_0.2_La_0.2_Pr_0.2_Sm_0.2_Y_0.2_)O_2−_ *_δ_*	Fluorite	Optoelectronics	Direct band gap of 2.03 eV	[217]
[(FeCoNiMn)_0.88_Na_0.12_]_3_O_4_	Spinel	Energy storage: Na‐ion anode	Average capacity decay per cycle of 0.069% at 1 A g^−1^; Oxygen vacancies improve cycling performance	[218]
(Zn_0.2_Ni_0.2_Mn_0.2_Fe_0.2_Ti_0.2_)_3_O_4_	Spinel	Energy storage: Li‐ion anode	Specific capacity of 480 mAh g^−1^; Improvements in capacity per cost	[24]
(MnCoFeNiCr)_3_O_4_	Spinel	Energy conversion: SOFC	Electrical conductivity of ≈3 S cm^−1^ at 700 °C; Removal of Cr results in decreased conductivity	[219]
(Mn_0.2_Fe_0.2_Ni_0.2_Mg_0.2_Cr_0.2_)_3_O_4_	Spinel	Catalysis: OER	Overpotential of 293 mV at 10 mA cm^−2^; Tafel slope of 46.5 mV dec^−1^; Addition of Mg improves catalytic performance	[220]
(MnFeCoNiCu)_3_O_4_	Spinel	Energy storage: Supercapacitor	Specific capacitance of 431 F g^−1^ at 0.5 A g^−1^; Coulomb efficiency of ≈92% after 10 000 cycles at 3 A g^−1^	[221]
(NiFeMnCuZn)_3_O_4_	Spinel	Catalysis: OER	Overpotential of 308 mV at 50 mA cm^−2^; Tafel slope of 54 mV dec^−1^; Significant oxygen vacancies improve OER activity	[222]

### 4.2 Laser Ablation and Inert Gas Condensation

Laser ablation and inert gas condensation (IGC) are two gas‐phase physical synthesis techniques that allow the production of nanomaterials with highly controlled particle size, morphology, and composition. Both approaches rely on the vaporization of bulk materials followed by nucleation and growth in a cooling gas environment, yet they differ in energy source, processing atmosphere, and scalability. While these methods are generally limited in throughput, they offer distinct advantages in purity, structural tunability, and access to metastable phases, making them valuable tools for developing HEMs with specialized functionalities.

Laser ablation employs a high‐intensity laser, often a pulsed Nd:YAG or excimer laser, focused onto the surface of a solid target, inducing rapid local heating, melting, and vaporization. The energetic interaction creates a transient plasma plume composed of atoms, ions, and clusters, which expands into a low‐pressure or inert atmosphere. Within this highly supersaturated vapor environment, nanoparticles nucleate and grow through gas‐phase condensation, followed by aggregation or sintering depending on plume density and thermal gradients. The particle size and morphology are strongly influenced by several interrelated parameters, including the laser wavelength, fluence, repetition rate, pulse duration, and the physical properties of the target. Substrate temperature also plays a critical role: insufficient heating often leads to the formation of amorphous particles, while optimal thermal conditions promote crystallinity.

The versatility of laser ablation has been demonstrated in the synthesis of diverse high‐entropy compositions, including CuCoMn_1.75_NiFe_0.25_, CrCoFeNiMn, and CrCoFeNiMnMo, particularly for electrocatalytic applications where high surface area, compositional homogeneity, and defect density are desirable.^[^ ^223^, ^224^, ^225^ ^]^ The ability to directly convert complex metallic targets into nanoscale oxides or alloys without chemical precursors offers a clean route to fabricate nanostructures such as core‐shell particles, heterostructures, and semiconductor quantum dots. In addition, laser ablation in reactive gases enables in‐situ oxidation or nitridation, tailoring the surface chemistry of the resulting particles. However, despite its advantages in precision and purity, the method is limited by target size, ablation rate, and scalability, making it most suitable for the preparation of small quantities of functional materials for thin‐film deposition, sensors, or catalytic studies.

Inert gas condensation (IGC), on the other hand, is a more classical method that operates under ultra‐high vacuum (UHV) conditions. In this technique, the source material is vaporized, typically through resistive heating, electron‐beam heating, or laser ablation, in a low‐pressure inert gas environment (e.g., He, Ar, Ne). The vapor then cools and condenses into nanoclusters through nucleation on the inert gas atoms, and these particles are subsequently collected on a cold substrate. Key process variables include the type and pressure of the inert gas, its flow rate, the evaporation rate of the metal, and the temperature of both the source and the collection surface. These parameters directly affect particle growth kinetics, collision frequency, and sintering.

IGC is particularly valued for its ability to produce highly pure, monodisperse nanoparticles with tailored sizes ranging from a few nanometers to tens of nanometers. Higher inert gas pressure or heavier gas species tend to enhance collision frequency and increase particle size, while increased flow rates and gas temperatures promote rapid quenching and smaller, less aggregated structures. As with laser ablation, IGC is well suited to preparing multicomponent alloys or metastable phases that may be difficult to achieve through equilibrium routes. Recent work has demonstrated the successful preparation of FeCoCrMoCBY and FeCoNiCrN nanoparticles using IGC, which were subsequently applied in electrocatalytic water‐splitting reactions.^[^ ^226^, ^227^ ^]^ These materials exhibit superior activity due to their fine particle size, uniform elemental mixing, and defect‐rich surfaces.

Overall, laser ablation and IGC provide powerful yet complementary approaches for the synthesis of high‐entropy nanomaterials under highly controlled, solvent‐free conditions. Both techniques enable the formation of clean, ligand‐free nanoparticles with unique structural features and high compositional complexity. While their scalability remains limited compared to wet‐chemical or spray‐based methods, their precision and tunability make them ideal platforms for exploring fundamental structure–property relationships in HEMs. Applications that benefit from high surface‐to‐volume ratios, narrow size distributions, and metastable phase retention, such as catalysis, energy harvesting, or optical sensing, gain the most from these advanced physical vapor techniques.

### 4.3 Functional Implications of Gas‐Phase and Aerosol‐Based Synthesis

**Figure 10:**
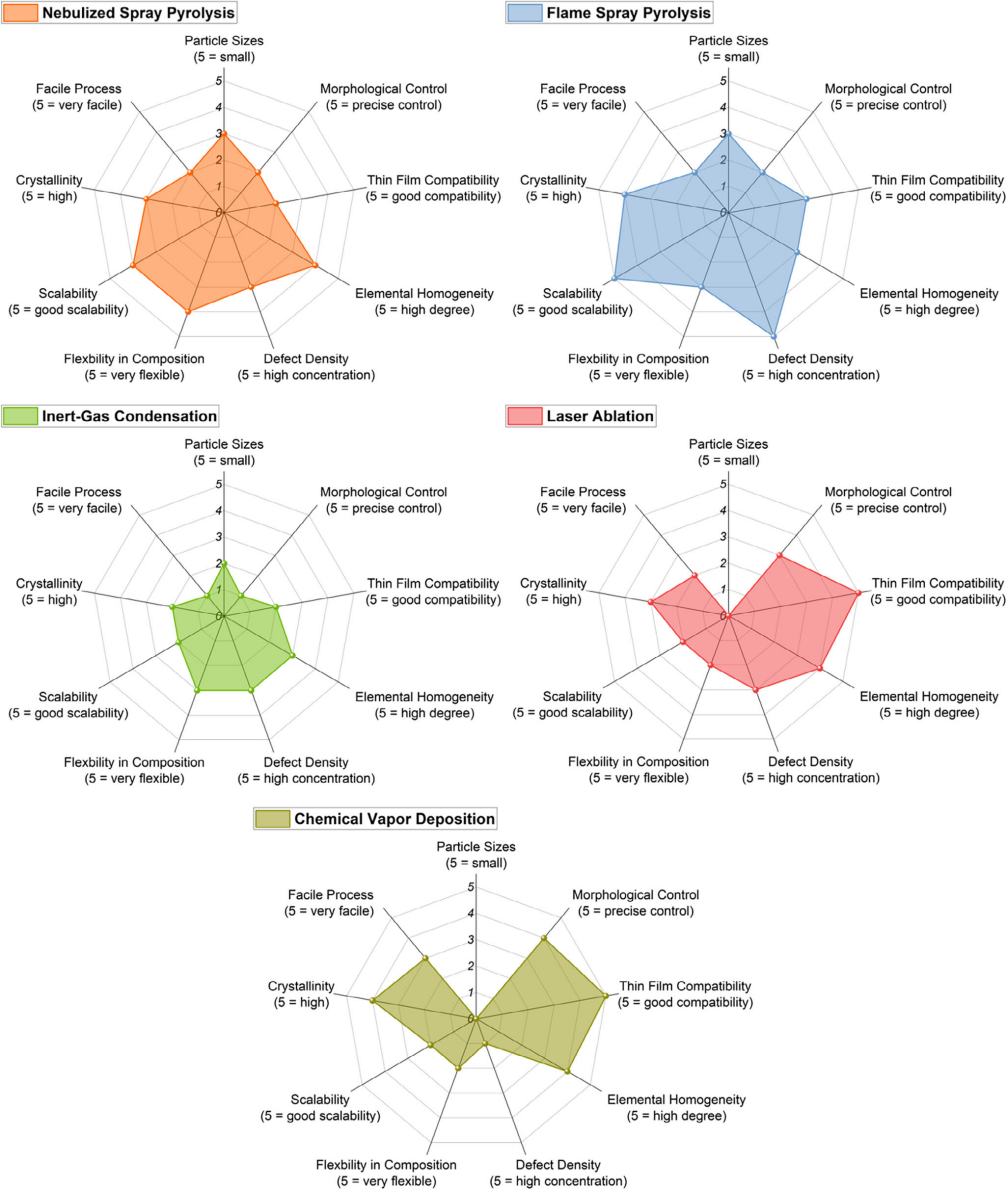
Radar charts for gas‐phase and aerosol‐based synthesis methods.

Gas‐phase and aerosol‐based synthesis techniques imprint a distinct set of morphological and physicochemical characteristics onto HEMs (**Figure** **10**), with strong downstream effects on their functional properties in catalytic, electrochemical, and thin‐film applications. These vapor‐based routes, such as CVD, NSP, IGC, and laser ablation, offer a high degree of control over size, crystallinity, and surface chemistry, while also enabling scalable production and integration into device architectures.

#### 4.3.1 Particle Size

Gas‐phase synthesis techniques typically yield tiny primary particle sizes, often in the nanometer range. Methods like IGC and laser ablation allow for exceptional control over the particle size due to their clean vapor‐phase condensation and rapid quenching environments. NSP also generates nanostructured particles, though maintaining sizes <100 nm remains a challenge, while FSP can synthesize tiny and monodisperse particles with narrow size distribution. CVS can tailor particle sizes through residence time and precursor volatility, allowing adjustment between thin‐film and particulate formation. These techniques overall offer better control compared to solid‐state methods and are often comparable or superior to solution‐based approaches in size control.

#### 4.3.2 Morphological Control

Morphology control in gas‐phase routes depends on temperature gradients, residence time, and the dynamics of nucleation versus sintering. For example, FSP produces nearly spherical, nanocrystalline particles due to rapid combustion and short residence times, while IGC and laser ablation can produce fractal, aggregated, or core‐shell morphologies depending on cooling rate and gas environment. CVS and NSP allow some tailoring of particle morphology through process parameters like droplet size, carrier gas type, and reactor temperature profile. However, while some morphology tuning is possible, these routes generally offer less morphological control than hydrothermal or sol‐gel methods.

#### 4.3.3 Thin‐Film Compatibility

Gas‐phase techniques are particularly well‐suited for thin‐film fabrication. CVS, especially in its aerosol‐assisted variant, and traditional CVD are widely used for film deposition and can be controlled between powder and film formation by adjusting gas flow and residence time. Laser ablation (in pulsed laser deposition setups, described in the thin‐film section) is a leading technique for producing high‐quality thin films of HEMs with tailored composition and thickness. NSP and FSP are less commonly used for films, but recent adaptations have enabled direct deposition of film‐like coatings on structured substrates. IGC is generally limited to nanoparticle production and is not film‐compatible.

#### 4.3.4 Elemental Homogeneity

Achieving elemental homogeneity is a core challenge in gas‐phase synthesis of HEMs, especially when multiple elements have varying vapor pressures or decomposition temperatures. Nonetheless, techniques like NSP and FSP mitigate this by localizing all cations within individual aerosol droplets, ensuring uniform decomposition and co‐nucleation. CVS benefits from optimized precursor chemistries, such as matched decomposition temperatures, enabling better mixing. IGC and laser ablation offer high homogeneity due to the vaporization of pre‐alloyed targets, though they are limited to materials that can be physically synthesized in bulk. Overall, compositional uniformity is achievable but highly sensitive to precursor and process design.

#### 4.3.5 Defect Density

The dynamic and often extreme environments in gas‐phase methods naturally introduce a variety of defects, such as oxygen vacancies, cation disorder, or stacking faults, which can be beneficial for applications like catalysis. FSP, for, produces oxygen‐deficient spinels that exhibit improved electrochemical performance. Laser ablation can tailor defect densities through energy and ambient pressure, while IGC yields defect‐rich particles due to the high supersaturation and rapid quenching. These methods are particularly suited for creating metastable, high‐energy phases with functional defects.

#### 4.3.6 Flexibility in Composition

Gas‐phase methods offer a wide but not unlimited compositional flexibility. Techniques such as NSP and CVS can accommodate a broad range of cations as long as suitable volatile precursors exist or can be combined in solution. FSP is more tolerant of non‐volatile precursors, expanding its compositional reach. Laser ablation and IGC are constrained by the need to fabricate bulk targets with the desired stoichiometry. Despite these limitations, multicomponent and even decenary (10‐element) HEMs have been synthesized using NSP, showcasing the potential of aerosol‐based gas‐phase methods for compositionally complex materials.

#### 4.3.7 Scalability

Aerosol‐based methods such as NSP and FSP are inherently scalable and already used in industrial settings for large‐scale production of oxides and other nanomaterials. Their continuous operation and compatibility with liquid precursors support high‐throughput synthesis. CVS also offers scalability, particularly with modular hot‐wall reactors. Conversely, IGC and laser ablation are batch processes with limited throughput and higher equipment costs, making them less suitable for large‐scale applications but ideal for research or high‐value functional materials.

#### 4.3.8 Crystallinity

Crystallinity in gas‐phase synthesized particles is strongly influenced by temperature and quenching rate. FSP yields nanocrystalline materials due to its high thermal budget and rapid cooling. NSP and CVS also produce crystalline particles, although post‐synthetic annealing may be needed to fully develop ordered phases, especially in complex systems. Laser ablation can yield either amorphous or crystalline nanoparticles depending on energy and substrate conditions. IGC tends to yield poorly crystalline particles unless additional energy is supplied post deposition. Crystallinity can thus be tailored through thermal control but often comes at the cost of particle sintering or aggregation.

#### 4.3.9 Process Simplicity

Gas‐phase methods range widely in process complexity. FSP and NSP are readily applicable once the precursor formulation is optimized and offer plug‐and‐play scalability. CVS and aerosol‐assisted CVD are more complex, requiring precise control over gas‐phase thermodynamics, but benefit from a high degree of tunability. Laser ablation and IGC involve high‐vacuum or pulsed laser systems, making them equipment‐intensive and less user‐friendly. While these methods offer unparalleled control over particle formation, they generally demand more technical infrastructure and expertise than solution‐based routes.

## 5 Thin‐Film Deposition Techniques for High‐Entropy Materials

**Figure 11:**
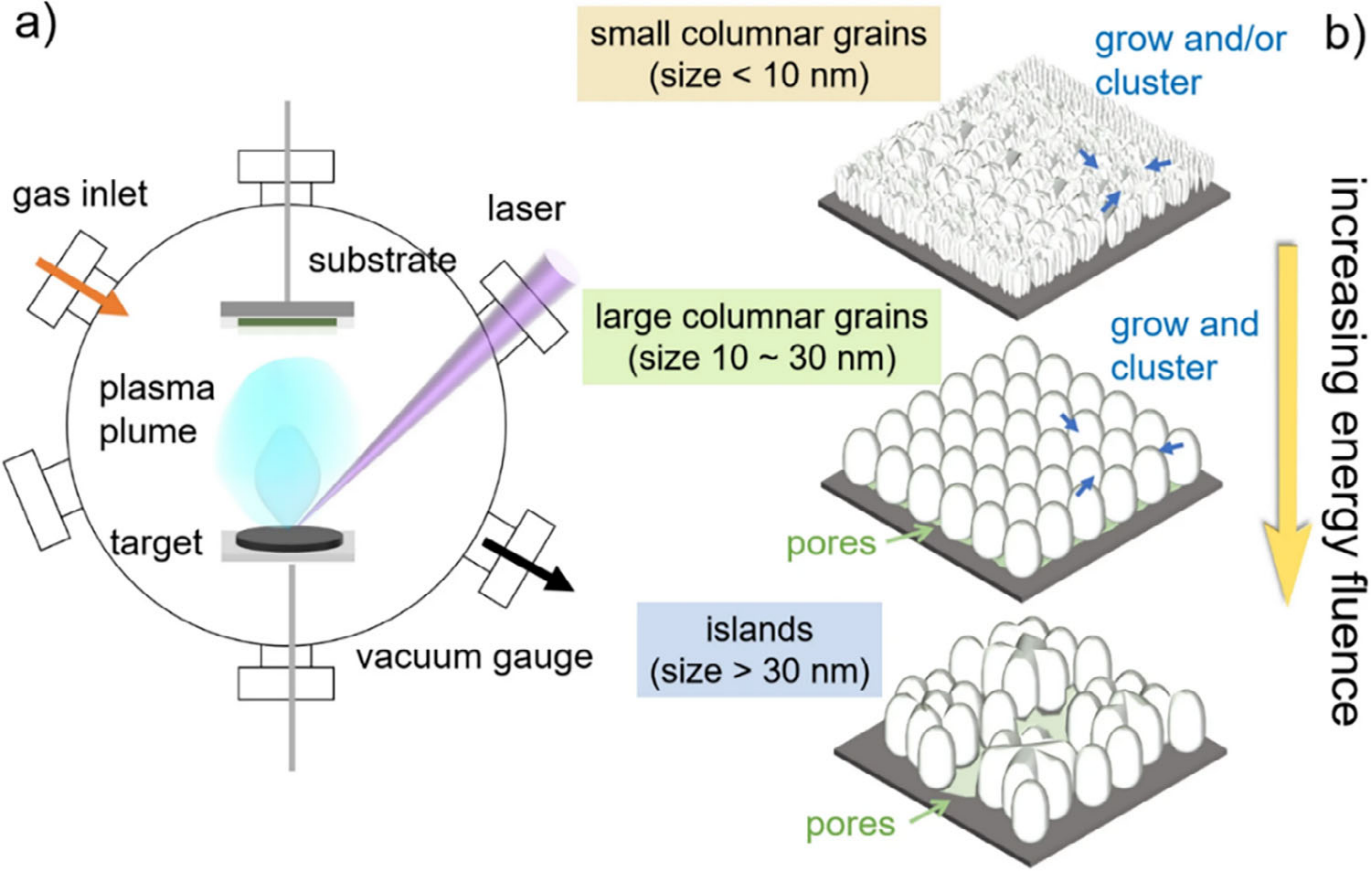
Schematic of (a) the principle of pulsed laser deposition and (b) the effect of increasing energy fluence on the 3D island structure of prepared thin films. Reproduced under terms of CC BY 4.0 license.^[^ ^265^ ^]^ Copyright 2022, Springer Nature.

Thin‐film deposition techniques are crucial for integrating HEMs into functional devices across electronics, photonics, catalysis, and energy storage. Traditional vacuum‐based methods such as sputtering, electron‐beam evaporation, and pulsed‐laser deposition (PLD) have been successfully employed to fabricate oxide, nitride, and carbide HEM thin films (**Figure** **11**).

Sputtering is widely used for depositing transparent conducting oxides, such as indium tin oxide, in applications like solar cells, OLEDs, and touchscreen devices. This approach has also been extended to HEOs, such as (AlCrNbTaTi)O_2_.^[^ ^228^ ^]^ Sputtering typically requires stoichiometric targets—either high‐entropy oxides^[^ ^229^ ^]^ themselves or metallic alloys subsequently oxidized during processing. Process conditions, including oxygen partial pressure and substrate temperature, play a critical role in tuning defect concentration and electrical conductivity. For example, Lin et al. demonstrated that adjusting the oxygen flow rate during deposition modulates the resistivity and optical band gap of (AlCrTaTiZr)O*_x_* films.^[^ ^230^ ^]^

Electron‐beam evaporation and PLD are preferred for materials with high melting or sublimation temperatures, such as aluminum or silicon oxides. These techniques enable the deposition of high‐quality oxide layers and complex multicomponent films. PLD, in particular, has proven versatile for the synthesis of complex HEOs, such as Ba(Zr_0.2_Sn_0.2_Ti_0.2_Hf_0.2_Nb_0.2_)O_3_, due to its ability to transfer the exact stoichiometry of a complex target to the film.^[^ ^231^ ^]^ However, due to differential vapor pressures and ablation thresholds of individual elements, stoichiometric fidelity must be carefully verified in HEO films.

The key advantages of PLD lie in its compositional tunability. By spatially arranging different targets or using combinatorial approaches, films with gradient compositions or discrete variations can be fabricated without producing a new HEO target for each composition. This approach has been successfully used to prepare thin films of (MgZnMnCoNi)O*_x_* and (CrFeMnCoNi)O*_x_*, where phase formation and electrical properties (e.g., spinel versus rock‐salt structure) were tailored via manganese content, consistent with DFT predictions.^[^ ^229^ ^]^ Further work on Mg_1/5_Co_1/5_Ni_1/5_Cu_1/5_Zn_1/5_O thin films grown using PLD has shown the dependence on the presence of Ni and Mg in obtaining a single‐phase thin film. However, Co, which is quite important in the formation of a single‐phase bulk HEO, exsolutes from the film at lower temperatures.^[^ ^232^ ^]^ PLD has been employed to synthesize high‐entropy ceramics for magnetic, dielectric, and piezoelectric applications.^[^ ^233^ ^]^ Thin films of spinel oxides have been studied for their magnetic properties,^[^ ^234^, ^235^, ^236^, ^237^ ^]^ while perovskite‐type HEO films such as (LaLuYGdCe)AlO_3_ were investigated for their optical and structural stability.^[^ ^238^, ^239^ ^]^ Ferroelectric and relaxor materials, including Ba(Ti_0.2_Sn_0.2_Zr_0.2_Hf_0.2_Nb_0.2_)O_3_,^[^ ^240^ ^]^ and lead‐based electrocaloric systems like Pb(Hf_0.2_Zr_0.2_Ti_0.2_Nb_0.2_Mn_0.2_)O_3_,^[^ ^241^, ^242^ ^]^ have shown promise for thermal regulation and energy harvesting applications.

Magnetic ordering in high‐entropy perovskite thin films has been reported for systems like Lu(Fe_0.2_Mn_0.2_Co_0.2_Cr_0.2_Ni_0.2_)O_3_ and La(Cr_0.2_Mn_0.2_Fe_0.2_Co_0.2_Ni_0.2_)O_3_, with weak ferromagnetism emerging from chemical disorder and local structural distortions.^[^ ^243^, ^244^, ^245^, ^246^, ^247^ ^]^ Additionally, rock‐salt, layered, and Ruddlesden‐Popper structures have been deposited as thin films via PLD, including high‐entropy nickelates for electrocatalysis and correlated electron phenomena.^[^ ^248^, ^249^, ^250^, ^251^, ^252^ ^]^

Magnetron sputtering has also gained traction for synthesizing HEMs based on nitrides, carbides, and borides. For instance, (Cr_0.35_Al_0.25_Nb_0.12_Si_0.08_V_0.20_)N coatings demonstrate enhanced mechanical hardness and oxidation resistance,^[^ ^253^, ^254^ ^]^ while (Zr_0.23_Ti_0.20_Hf_0.19_V_0.14_Ta_0.24_)B_2_ exhibits excellent thermal and mechanical performance.^[^ ^255^ ^]^ Transition metal carbides and diborides have also been investigated for charge storage,^[^ ^256^ ^]^ catalysis, and thermal barrier applications. Furthermore, solution‐processable high‐entropy sulfides, such as (CuZnCoInGa)S, have been deposited via CVD.^[^ ^211^ ^]^

As device fabrication trends move toward printing, coating, and roll‐to‐roll processing, wet‐chemical routes become increasingly relevant. Two principal methods for oxide film deposition from solution are precursor‐based routes and nanoparticle dispersions (slurries). While simple binary oxides such as ZnO and WO_3_ can be deposited from acetates or alkoxides at low temperatures (100–150 °C),^[^ ^257^, ^258^ ^]^ extending this to complex HEOs remains challenging.

The main difficulty lies in synchronizing the decomposition behavior of precursors with varying thermal stabilities. Premature decomposition or delayed reactivity may lead to phase segregation or poor crystallinity. Nevertheless, sol‐gel routes have demonstrated potential for fabricating HEO thin films, including Ba(Zr_0.2_Sn_0.2_Ti_0.2_Hf_0.2_Nb_0.2_)O_3_,^[^ ^259^ ^]^ and Bi‐Na‐K‐La‐Sr titanates with ferroelectric functionality.^[^ ^260^, ^261^ ^]^ Volume shrinkage during gel‐to‐oxide conversion can result in cracking—especially in thick films—but can be mitigated via slurry‐based processing, which relies on preformed oxide powders and densification through high‐temperature sintering.

Dip‐coating and spin‐coating are the most common wet‐chemical deposition methods.^[^ ^233^, ^262^, ^263^ ^]^ Although still rare in the HEM field, these techniques offer cost‐effective and scalable routes for device integration. Initial reports of epitaxially grown high‐entropy nickelate films from acetate‐based sol‐gels with controlled orientation are promising for tailoring crystal growth and anisotropic properties.^[^ ^264^ ^]^

Despite being less mature than physical vapor methods, solution‐processed HEM films hold great promise. They allow low‐cost synthesis, compositional flexibility, and potential compatibility with soft substrates, flexible electronics, and inkjet printing, key for future applications in wearables, energy‐harvesting devices, and sensors.

### 5.1 Functional Implications of Thin‐Film Deposition Techniques

**Figure 12:**
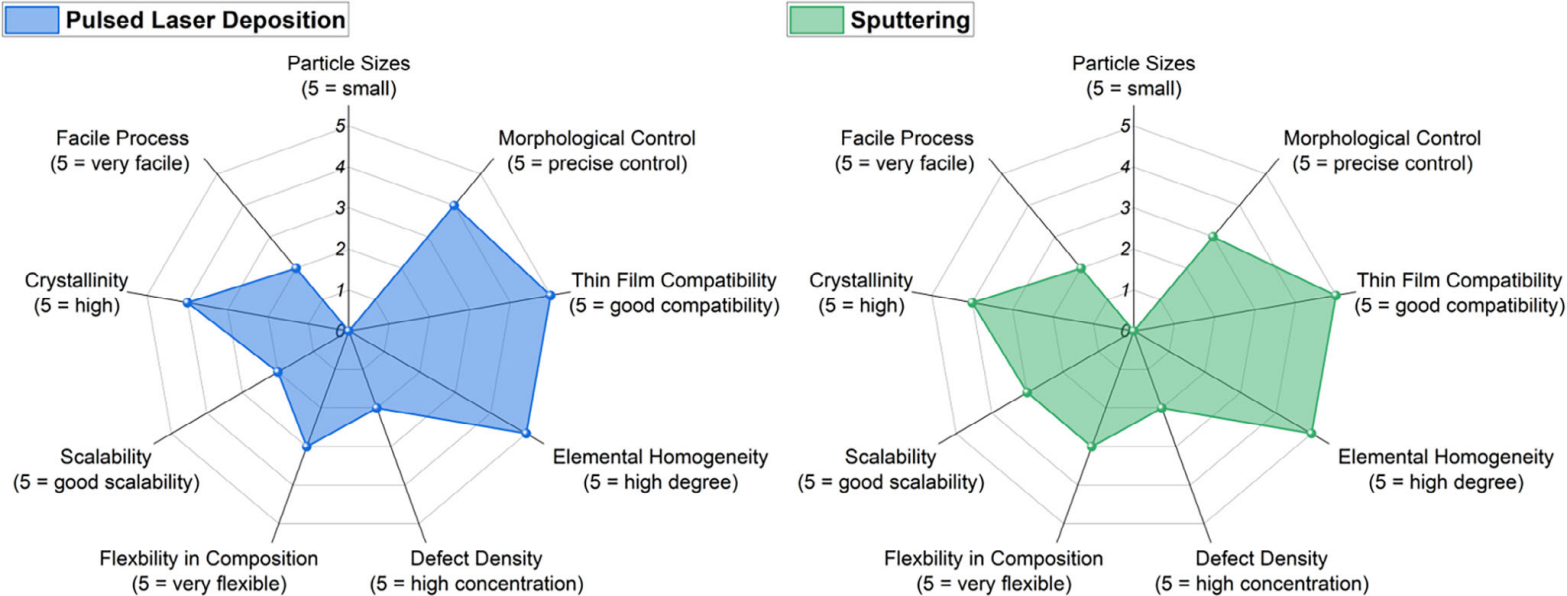
Radar charts for thin‐film deposition techniques.

Thin‐film deposition techniques uniquely influence the microstructure, defect landscape, and functional properties of HEMs (**Figure** **12**), often dictating their suitability for advanced electronic, magnetic, catalytic, and energy applications. Understanding these synthesis‐dependent characteristics is critical for optimizing device performance and guiding materials design.

#### 5.1.1 Particle Size

Thin‐film deposition techniques afford precise control over film thickness at the nanometer level, which is crucial for tailoring the functional properties of HEMs. Physical vapor deposition (PVD) methods such as magnetron sputtering, electron‐beam evaporation, and PLD enable the fabrication of films with controlled grain sizes, often ranging from a few nanometers to several tens of nanometers. PLD, in particular, can produce epitaxial films with ultra‐smooth surfaces and uniform thicknesses down to atomic layers, facilitating investigations of size‐dependent phenomena. In contrast, solution‐based deposition methods (e.g., sol‐gel, dip‐coating, spin‐coating) rely on precursor chemistry and thermal treatment to define particle size and film thickness, typically resulting in less precise control and broader particle size distributions before post‐deposition annealing.

#### 5.1.2 Morphological Control

The ability to control thin‐film morphology critically influences surface reactivity, charge transport, and mechanical stability. PVD techniques yield dense, continuous films with uniform morphology and minimal porosity. Substrate temperature, ambient gas composition, and deposition rate are key parameters modulating nucleation density, grain growth, and film texture. For example, oxygen partial pressure during sputtering can affect grain‐boundary density and film roughness in oxide HEMs. Solution‐processed films exhibit more complex morphologies due to solvent evaporation dynamics and precursor decomposition kinetics, which can induce porosity, cracking, or non‐uniform thickness. However, slurry‐based deposition and advanced sol‐gel chemistries have shown promise in producing crack‐free, dense films with improved morphological uniformity.

#### 5.1.3 Thin‐Film Compatibility

PVD techniques are compatible with a broad range of substrates, including crystalline wafers, metals, and transparent conducting oxides (TCOs), due to the high‐energy nature of deposition and substrate heating capabilities. This facilitates integration into microelectronic and optoelectronic devices. Solution‐based processes expand substrate options further to include flexible polymers and temperature‐sensitive materials, enabling novel applications in flexible and wearable technologies. However, substrate‐film interactions, thermal expansion mismatches, and chemical compatibility require careful management to prevent delamination or cracking, especially in thick or multilayer films.

#### 5.1.4 Elemental Homogeneity

Achieving compositional uniformity is paramount for high‐entropy thin films to realize their synergistic effects. Sputtering and PLD provide near‐stoichiometric transfer of multicomponent targets to substrates, ensuring excellent elemental homogeneity, although discrepancies can arise due to differential sputtering yields or ablation thresholds among constituent elements. Careful process calibration and target design mitigate these effects. Solution‐based routes must address the challenges of precursor compatibility, varying thermal decomposition profiles, and phase segregation risks. Recent advances in sol‐gel precursor design and slurry homogenization have improved elemental uniformity, but maintaining homogeneity in thick films remains challenging due to diffusion limitations during annealing.

#### 5.1.5 Defect Density

Defect engineering is a double‐edged sword in HEM thin films. Controlled defect introduction can enhance properties such as catalytic activity or ionic conductivity, whereas excessive defects degrade performance. PVD techniques allow tailoring of defect density by adjusting parameters like oxygen partial pressure, substrate bias, and temperature. For example, sputtered oxide films exhibit oxygen vacancy concentrations correlated to deposition atmosphere, which influence conductivity and optical absorption. Solution routes inherently introduce higher defect densities due to structural disorder from incomplete densification and residual stresses, but controlled annealing and precursor chemistry optimization can reduce defect levels. Detailed characterization is necessary to balance beneficial and detrimental defect populations.

#### 5.1.6 Flexibility in Composition

One of the main advantages of PLD is its exceptional compositional flexibility, enabling combinatorial deposition from multiple targets to generate compositional gradients or libraries without fabricating new targets. This facilitates rapid exploration of phase space in high‐entropy systems. Magnetron sputtering also supports compositional tailoring via alloy targets or co‐sputtering from elemental sources. Solution‐based methods allow for facile adjustment of precursor ratios, offering a low‐cost approach to composition variation. However, complex precursor chemistries and differing thermal properties can complicate phase stability and elemental mixing in these wet‐chemical routes.

#### 5.1.7 Scalability

While magnetron sputtering and electron‐beam evaporation are mature, industry‐compatible techniques for large‐area thin‐film fabrication, their cost and vacuum requirements can limit accessibility. The inherently small deposition area of PLD and limited target size restrict throughput, confining its use mainly to laboratory‐scale research. Conversely, wet‐chemical deposition methods, including dip‐coating and spin‐coating, are promising for scalable, cost‐effective manufacturing and are compatible with roll‐to‐roll and printing technologies vital for flexible electronics and wearable devices. Nevertheless, challenges remain in achieving reproducible film quality, uniformity, and adhesion at industrial scales.

#### 5.1.8 Crystallinity

High crystallinity is essential for optimizing electronic, magnetic, and optical properties of HEM thin films. PVD techniques generally yield highly crystalline films, owing to energetic species and elevated substrate temperatures facilitating adatom mobility and epitaxial growth. For instance, PLD‐grown perovskite and spinel HEO films often exhibit well‐defined crystallographic orientations and phase purity. Conversely, solution‐processed films typically require high‐temperature annealing to induce crystallization, with the risk of incomplete phase formation or secondary phase development due to uneven heating or precursor decomposition heterogeneity. The trade‐off between crystallinity and process temperature remains a significant consideration in solution‐based methods.

#### 5.1.9 Process Simplicity

PVD methods demand sophisticated vacuum equipment, high‐purity targets, and precise process control, entailing significant capital and operational costs. Nevertheless, they deliver films with superior uniformity and functional properties essential for advanced applications. In contrast, solution processing offers a simpler, low‐cost alternative with minimal equipment requirements. However, the complexity of precursor chemistry, the need for multi‐step thermal treatments, and potential film defects complicate process reproducibility. Advances in precursor formulation and processing strategies are gradually addressing these limitations, enhancing the practicality of solution‐processed HEM thin films.

## 6 High‐Throughput Synthesis Methods for HEM Discovery

The discovery and rapid optimization of new materials are pivotal for advancing technologies across diverse fields. Traditionally, materials science has relied on a “trial and error” approach guided by prior knowledge, which reduces the need for extensive testing of countless material and synthesis parameter combinations (e.g., calcination temperature, time). However, this rational design approach becomes increasingly limited when dealing with complex systems such as multielement doping or HEMs. The synthesis complexity of HEMs, characterized by the simultaneous variation of several elements, grows exponentially, complicating the identification and optimization of ideal material compositions from millions of possibilities. This complexity leads to only a handful of optimized candidates suitable for practical applications. One promising route to accelerate this process is the use of theoretical models and simulations capable of predicting properties such as electrochemical activity and phase stability.^[^ ^266^ ^]^

Recent advances in computational materials science have introduced first‐principles methods that predict the intricate interactions between composition, structure, and properties of HEMs.^[^ ^267^, ^268^, ^269^, ^270^, ^271^, ^272^, ^273^ ^]^ Additionally, high‐throughput computational screening has made significant strides in predicting phase properties in multicomponent compositions. These methods utilize empirical guidelines derived from existing HEM studies or apply phase diagram calculation techniques (CALPHAD) to efficiently narrow the compositional parameter space, allowing the screening of millions of potential compositions.^[^ ^56^, ^274^, ^275^ ^]^ Despite these powerful tools, computational models primarily address bulk equilibrium thermodynamics and often struggle to fully capture the properties of nanoscale HEMs or thin films, where kinetic effects and non‐equilibrium phases play a critical role. Consequently, comprehensive experimental synthesis, characterization, and performance testing of diverse compositions remain indispensable.^[^ ^276^ ^]^

**Figure 13:**
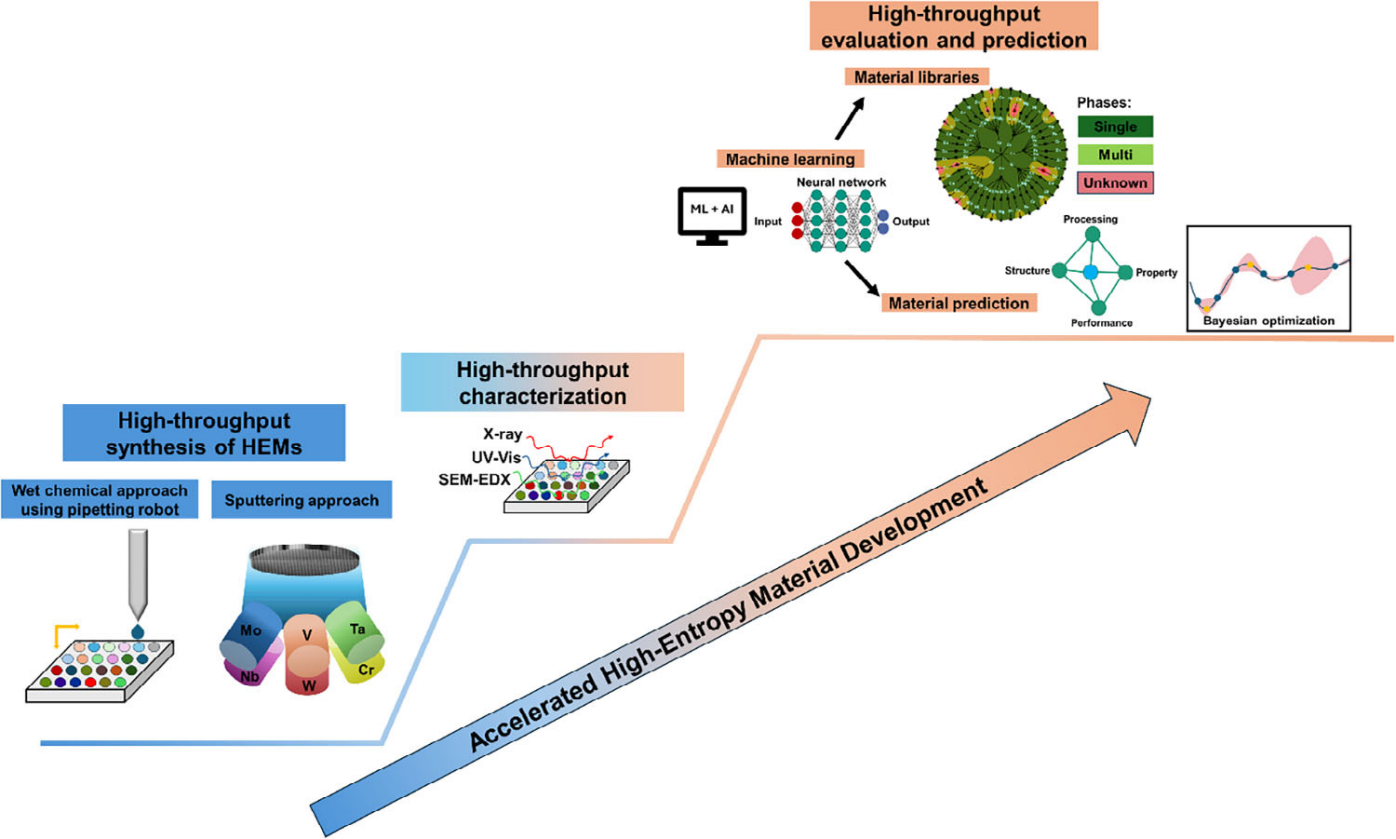
Scheme of a high‐throughput approach for the preparation of HEMs libraries.

Combinatorial, automated, and high‐throughput experimental methods offer promising strategies for rapidly screening a vast range of material compositions (**Figure** **13**). These approaches facilitate the creation of material libraries (MLs), providing valuable data that can train machine learning (ML) and artificial intelligence (AI) algorithms, potentially reducing the need for extensive synthesis and characterization cycles and accelerating materials development.^[^ ^276^, ^277^ ^]^ However, terminology in the field can be ambiguous: “combinatorial” methods often refer to experiments combining different elements, solvents, or additives to vary parameter types, whereas “high‐throughput” relates more to the volume and systematic variation of parameters such as composition, temperature, or pressure. Because many studies blend these approaches, this review adopts the combined term “combinatorial/high‐throughput methods” to describe simultaneous, parallel synthesis of multiple samples. This contrasts with fully automated, robot‐assisted synthesis where samples are prepared sequentially.^[^ ^278^ ^]^ Here, we focus on both automated and combinatorial/high‐throughput synthesis approaches tailored specifically to HEMs, distinguishing between thin‐film and powder/bulk material syntheses based on technique.

## 7 Thin‐Film and Related Techniques

Combinatorial/high‐throughput methods such as sputtering enable rapid exploration of HEM composition space by generating diverse materials in parallel. This approach directly addresses the challenge of screening the enormous compositional landscape inherent to HEMs, where near‐equimolar combinations yield complex property variations. Two primary thin‐film techniques for creating gradient material libraries are (co)evaporation and (co)sputtering.^[^ ^279^ ^]^ These material libraries, sometimes called gradient libraries, facilitate systematic property mapping over wide compositional ranges.

## 8 Sputtering

Sputtering and co‐sputtering are mature techniques for depositing continuous multilayers or atomically mixed films. A cathode is bombarded with high‐energy ions from a plasma, ejecting atoms from the target that deposit on substrates under ultra‐high vacuum conditions. Magnetron sputtering improves deposition rates and plasma density by confining secondary electrons with magnetic fields, enabling more efficient ionization. Different magnetron types—direct current (DC) and radio frequency (RF)—suit various material classes; DC is cost‐effective for conductive targets, while RF can handle insulating materials.

In combinatorial sputtering, wedge‐shaped layers are deposited by gradually retracting movable shields or co‐sputtering from multiple targets with shutters at specific angles, allowing atomic‐scale mixing of multiple elements in gradient form.^[^ ^280^ ^]^ The sputtering atmosphere (Ar, O_2_, N_2_, or mixtures) further tunes film composition, enabling deposition of metals, oxides, or nitrides.^[^ ^281^, ^282^, ^283^ ^]^ Early non‐HEM combinatorial sputtering experiments date back to the 1960s,^[^ ^284^, ^285^, ^286^ ^]^ with more recent studies extending these techniques to complex concentrated solid solutions (CCSS) and HEMs.

Notable examples include the creation of over 1000 quinary metallic composition libraries (Co‐Cr‐Fe‐Mo‐Ni) by systematically varying target positions by Schumacher et al.,^[^ ^287^ ^]^ and the exploration of 2052 compositions in Ru‐Rh‐Pd‐Ir‐Pt HEAs by Banko et al.,^[^ ^276^ ^]^ both correlating composition with electrocatalytic properties. Gradient thin films of 169 HEA systems were studied by Schweidler et al. with respect to hardness and electrical resistance.^[^ ^288^ ^]^ Further advancements include coating complex substrates like polystyrene microspheres and alloy particles with HEM thin films, expanding applications to catalysis and nanoparticle synthesis by co‐sputtering into ionic liquids.^[^ ^289^, ^290^ ^]^

Non‐metallic HEM thin films such as oxides and nitrides can be deposited by reactive sputtering using mixed gases. For example, spinel‐type Cantor‐alloy oxides (CoCrFeMnNi)_3_O_4_ thin films were prepared by sputtering under Ar/O_2_ atmospheres,^[^ ^229^, ^291^ ^]^ while nitrides like HfNbTiVZrN and (AlCrTiZrV)N_x_ were synthesized with compositional gradients to study mechanical and microstructural properties.^[^ ^292^, ^293^ ^]^ Variations in reactive gas flows directly influenced phase formation, crystallinity, hardness, and elastic modulus, highlighting the tunability of sputtering parameters for functional optimization.^[^ ^253^, ^294^ ^]^

## 9 Automated Powder/Bulk Material Synthesis

Automated platforms integrating synthesis, characterization, and testing have emerged for bulk and powder HEMs.^[^ ^30^, ^295^, ^296^, ^297^, ^298^, ^299^, ^300^, ^301^, ^302^, ^303^, ^304^, ^305^, ^306^ ^]^ Typically, these involve robotic pipetting, powder dispensing, or inkjet printing to prepare samples with systematically varied compositions. Pipetting robots mix aqueous precursor solutions in programmed ratios, often inducing gel formation via co‐precipitation or sol‐gel methods, followed by drying and calcination. While literature is still limited, studies by Velasco et al.^[^ ^306^ ^]^ and Kumbhakar et al.^[^ ^30^ ^]^ have applied automated co‐precipitation to rare‐earth‐based fluorite‐type HEMs, exploring phase stability and oxygen vacancy distributions. Sol‐gel approaches appear particularly promising due to their solution processability and controlled chemistry.

Inkjet printing, a non‐contact, reproducible technique, is increasingly used for rapid material fabrication. Modified printers enable precise mixing of metal species, solvents, and stabilizers to produce nanoparticle dispersions, which are then printed and calcined. Although inkjet printing of HEMs is nascent, analogous high‐throughput syntheses have been reported for multicomponent oxides like Mg_x_Ni_y_Cu_z_TiO_w_ and Fe_v_C_w_NiTi_y_O_z_.^[^ ^304^, ^307^ ^]^

In solid‐state synthesis, automated powder dosing systems, though underutilized in ceramics, offer promising avenues for high‐throughput synthesis and additive manufacturing methods such as directed energy deposition (DED). These systems can accurately weigh, mix, press, and sinter powders, enabling rapid sample preparation.^[^ ^308^, ^309^ ^]^ DED, utilizing plasma, laser, or electron beams, can melt multiple powder feedstocks to produce compositionally tailored materials layer‐by‐layer, presenting opportunities for HEM fabrication via additive manufacturing.

### 9.1 Functional Implications of High‐Throughput and Automated Synthesis Methods

The integration of high‐throughput and automated synthesis techniques has fundamentally transformed the exploration and optimization of HEMs. These methods address the immense compositional complexity inherent to HEMs by enabling rapid, parallel synthesis of large compositional libraries, significantly accelerating the identification of promising candidates. Thin‐film combinatorial techniques such as magnetron sputtering and co‐sputtering provide a powerful platform to systematically vary elemental ratios across gradients, facilitating detailed mapping of phase formation, microstructure, and functional properties like electrocatalytic activity, mechanical hardness, and electronic conductivity. This spatially resolved synthesis, coupled with rapid characterization, enables efficient screening of vast chemical spaces that would be infeasible through conventional serial experimentation.

Automated powder and bulk synthesis platforms complement thin‐film methods by extending high‐throughput capabilities to bulk materials, which are critical for practical applications requiring higher material volumes or specific morphologies. Robotic pipetting and automated solid‐state powder processing enable precise control over precursor mixing, gelation, calcination, and sintering steps, ensuring reproducibility and scalability. Inkjet printing and additive manufacturing approaches offer further versatility by allowing compositional tailoring and complex shaping in powder‐based materials. These developments pave the way for accelerated discovery workflows that combine precise compositional control with scalable production.

However, despite these advances, the sheer scale of the HEM compositional landscape necessitates a close integration of experimental synthesis with computational strategies. Machine learning and Bayesian optimization frameworks synergize with high‐throughput experimentation to efficiently navigate the vast parameter space, reducing experimental burden while enhancing predictive accuracy. This iterative feedback loop between synthesis, characterization, and data‐driven modeling enables targeted optimization of key material properties such as phase purity, catalytic performance, mechanical robustness, and electronic behavior.

Collectively, the convergence of high‐throughput thin‐film deposition, automated bulk synthesis, and advanced computational tools represents a paradigm shift in materials discovery. It transforms the traditional trial‐and‐error approach into a data‐driven, accelerated design process, essential for unlocking the full potential of HEMs across energy, catalysis, electronics, and structural applications.

## 10 Challenges and Future Perspectives

Despite advances, the immense combinatorial space of HEMs, driven by element permutations and stoichiometric variations, poses a fundamental bottleneck: the number of potentially interesting compositions far exceeds the capacity of even high‐throughput experimental methods. Purely experimental exploration is therefore infeasible. To address this, high‐throughput experimentation must be integrated with computational techniques. Conventional methods such as density functional theory (DFT) are often computationally prohibitive for HEMs and limited in predictive accuracy. Machine learning offers a promising alternative to uncover complex structure‐property relationships but requires large, high‐quality datasets often beyond current experimental generation capabilities.

Bayesian optimization provides an efficient solution by iteratively guiding experiments to the most promising regions of composition space, drastically reducing required sample numbers while maintaining predictive power. Friederich et al. demonstrated this approach in optimizing non‐noble oxygen evolution catalysts, suggesting its applicability for HEMs.^[^ ^310^ ^]^ Such closed‐loop frameworks combining high‐throughput synthesis, characterization, and machine learning could revolutionize accelerated discovery and targeted optimization of high‐entropy materials.

## 11 Summary

In summary, the choice of synthesis route plays a decisive role in determining the structure, morphology, and ultimately the functional properties of HEMs. Beyond the intrinsic complexity of elemental interactions within multicomponent systems, the synthesis method governs key physical parameters such as particle size, morphology, defect density, and phase purity, all of which critically influence performance in subsequent applications.

Solid‐state and mechanochemical methods generally yield larger particles with relatively low surface area but often high crystallinity, suitable for bulk structural applications or as precursors for further processing. By contrast, wet‐chemical approaches such as sol‐gel or co‐precipitation enable finer control over particle size and homogeneity, producing nanoparticles with tailored morphology and enhanced compositional uniformity, which are particularly advantageous for catalytic or electrochemical applications where surface effects dominate. Pyrolytic and aerosol‐based syntheses further allow continuous and scalable production of nanomaterials with controllable particle agglomeration and porosity, important for energy storage and sensing technologies.

Thin‐film deposition techniques, especially pulsed laser deposition and magnetron sputtering, provide unparalleled control over compositional gradients, crystallographic orientation, and defect engineering in high‐entropy oxide films. This precise tailoring of microstructure at the nanoscale opens new frontiers in electronics, optics, and magnetism, where subtle variations in lattice strain, vacancy concentrations, or doping levels can drastically alter dielectric, ferroelectric, or catalytic behavior. The ability to create gradient libraries via combinatorial sputtering also accelerates discovery by enabling rapid screening of vast compositional spaces within a single substrate.

Automated and high‐throughput synthesis methods further extend these capabilities by integrating robotics and advanced analytics, allowing systematic exploration of multidimensional composition‐process‐property landscapes. These approaches are essential to overcome the combinatorial explosion inherent in HEM systems, facilitating the identification of optimal material candidates for specific applications such as electrocatalysis, piezoelectrics, or energy storage. Combined with machine learning and data‐driven feedback loops, they promise to revolutionize the pace and efficiency of HEM research.

Ultimately, the synthesis method chosen determines not only the achievable phase stability and compositional complexity but also the defect structure, particle size distribution, and microstructural features that define the properties of a material. Understanding these interdependencies is crucial to designing HEMs with tailored performance for catalytic activity, mechanical robustness, electrical conductivity, optical transparency, or thermal stability. As research advances towards compositionally complex thin films and scalable powder synthesis, a rational and methodical selection of synthesis strategies will be pivotal in unlocking the full potential of high‐entropy materials across diverse technological fields. With rapid advancements in synthesis technologies and the growing integration of automated, data‐driven approaches, we stand at the forefront of a new era in high‐entropy materials research. These diverse methods not only enable precise design of complex multi‐element systems but also open up exciting avenues for sustainable, high‐performance, and innovative applications ranging from energy technologies to advanced electronics. This interdisciplinary progress promises to transcend the boundaries of conventional materials science and paves the way for transformative technologies that will significantly impact our future world.

## Conflict of Interest

The authors declare no conflict of interest.
